# Glycerol and Glycerol-3-Phosphate: Multifaceted Metabolites in Metabolism, Cancer, and Other Diseases

**DOI:** 10.1210/endrev/bnaf033

**Published:** 2025-09-10

**Authors:** S R Murthy Madiraju, Elite Possik, Fahd Al-Mulla, Christopher J Nolan, Marc Prentki

**Affiliations:** Department of Nutrition, Biochemistry and Molecular Medicine, University of Montreal, Montreal, QC, Canada H2X 0A9; Montreal Diabetes Research Center, Centre de Recherche du Centre Hospitalier de l’Université de Montréal (CRCHUM), Montreal, QC, Canada H2X 0A9; DREAM-CV Lab, Research Institute of the McGill University Health Center (RI-MUHC), Montreal, QC, Canada H4A 3S5; Translational Research Department, Dasman Diabetes Institute, Kuwait City 15462, Kuwait; School of Medicine and Psychology and John Curtin School of Medical Research, College of Health and Medicine, Australian National University, the Canberra Hospital, Canberra, ACT 2605, Australia; Department of Nutrition, Biochemistry and Molecular Medicine, University of Montreal, Montreal, QC, Canada H2X 0A9; Montreal Diabetes Research Center, Centre de Recherche du Centre Hospitalier de l’Université de Montréal (CRCHUM), Montreal, QC, Canada H2X 0A9

**Keywords:** glycerol metabolism, glycerol-3-phosphate, glycerol shunt, glycerolipid cycle, glyceroneogenesis, energy metabolism

## Abstract

Glycerol and glycerol-3-phosphate (Gro3P) are key metabolites at the intersection of carbohydrate, lipid, and energy metabolism. Their production and usage are organismal and cell-type specific. Glycerol has unique physicochemical properties enabling it to function as an osmolyte, protein structure stabilizer, and an antimicrobial and antifreeze agent, important to the preservation of many biological functions. Glycerol and Gro3P are implicated in many physiological and disease processes relating to energy metabolism, thermoregulation, hydration, skin health, male fertility, aging, and cancer. Glycerol has countless applications in the food, pharmaceutical, and cosmetics industries. It is used as a sweetener, preservative, thickening agent, humectant, osmolyte, and cryoprotectant. It is widely used in skin and wound care products, laxatives, in cell and tissue preservation, and in medicines for numerous conditions. Here, we review the multiple uses and functions of glycerol and Gro3P and associated transporters, enzymes, and target genes in health, senescence, and disease. We discuss the evidence that glycerol may be present at much higher levels in tissues and cells than in the blood. We bring particular focus to the newly identified glycerol shunt in the direct formation of glycerol independent of lipolysis and as a pathway allowing cells to adapt to various stresses. Relevant to chronic metabolic diseases, cancer and aging, glycerol and Gro3P present important translational implications and thus warrant much more attention.

## Essential Points

- Glycerol, a 3-carbon polyol, is generally viewed as a relatively inert molecule useful in various industrial applications and is the product of lipolysis, acts as a gluconeogenesis substrate, and is the backbone for glycerolipids
- In addition to lipolysis, glycerol in mammalian cells is also directly generated from glucose-derived glycerol-3-phosphate by the newly identified enzyme glycerol-3-phosphate phosphatase (G3PP; gene name *PGP*)
- G3PP operates a novel metabolic pathway, the glycerol shunt, that shunts glucose-derived carbons away from glycolysis and lipogenesis, particularly under conditions of excess glucose and hypoxia
- The glycerol shunt acts as a “glucose excess detoxification machine” and as a “reductive stress security valve” and influences glucose, lipid, redox, and energy metabolism, particularly when glycerol-3-phosphate is elevated
- Glycerol and/or glycerol-3-phosphate and associated metabolic enzymes and aquaglyceroporins that transport glycerol in and out of cells are implicated in biological processes such as energy metabolism, thermoregulation, skin hydration, osmotic regulation, protection from cold, heat and oxidative stress, glycation, protein chaperone, sperm health and male fertility, as well as cell senescence
- There is evidence of much higher concentrations of glycerol within the cells in comparison to plasma, the significance of which needs to be understood
- Variations in glycerol levels and variants of genes encoding glycerol and glycerol-3-phosphate metabolic enzymes and transporters are implicated in many diseases such as obesity, cardiometabolic diseases, skin disorders, brain edema, citrin deficiency, several cancers, as well as in aging

It was more than 200 years ago in 1783 that glycerol was discovered during the distilling of olive oil in the presence of lead oxide by the Swedish pharmacist Carl Wilhelm Scheele, who called it the “sweet principle of fat.” Later in 1811, this substance was shown by the French chemist Michel-Eugène Chevreul to be a constituent of fats in a combined form with fatty acids. He named it glycerine from the Greek word “glykós” for sweet, followed by “ine” related to substances associated with life (1, 2). Since these foundational discoveries, advances in knowledge on the chemical, industrial, biochemical, and medical aspects of glycerol have been immense (3), and yet, as highlighted in this review, many gaps remain in our understanding of the multiple roles of this seemingly simple 3-carbon polyol in health and disease.

Traditionally, glycerol in mammals has been viewed as a relatively inert molecule, important as the backbone of glycerolipids (GLs), including triglycerides (TGs) for energy storage and phosphoglycerolipids in cell membranes, and as a substrate for gluconeogenesis during fuel mobilization via lipolysis during fasting (3). However, much more about glycerol has been learned through studying its roles in microorganisms and small multicellular organisms (eg, *C elegans* and yeast), particularly on how it can protect cells of these organisms from a variety of external stresses, such as heat, cold, and oxidative/reductive and hyperosmotic conditions (4). Improved understanding of glycerol metabolism in mammals has also come from studying how glycerol enters and exits specific cell types via aquaglyceroporins (AQPs) (5), and the relatively recent discovery of the glycerol shunt that is implicated in excess nutrient detoxification and several additional functions (6, 7). Hence, greater attention is currently being focused on glycerol's roles in many biological processes and diseases ranging from cardiometabolic diseases, skin disorders, male infertility, aging, and some cancers (8-12).

Despite glycerol's increasingly recognized importance in health and disease processes, it comes as a surprise that its diverse roles in physiological and pathological processes have so far not been reviewed in a comprehensive manner. This review is intended to provide that broad overview of the current state of knowledge on glycerol in health, in response to stress, and in disease, and to indicate the gaps in knowledge yet to be filled. Further, possible therapeutic avenues targeting key players in glycerol metabolism, such as by enhancing glycerol-mediated cytoprotection and/or activating glycerol-related nutrient detoxification pathways for various diseases, is discussed.

## Physicochemical Properties of Glycerol Underpinning Its Current Use in Food and Medical Therapies

**Figure 1.**
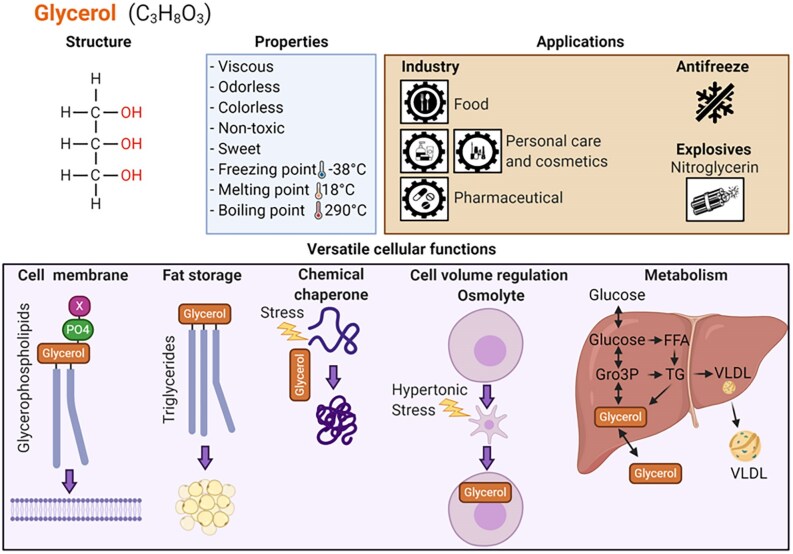
Physicochemical properties, applications, and cellular functions of glycerol. Glycerol is a 3-carbon triol with a molecular weight of 92.09. It is a viscous, sweet-tasting liquid at room temperature without color and odor and with its freezing, melting, and boiling points much different from those of water. It has multiple applications in industry, as an antifreeze and in the manufacture of the explosive nitroglycerin. In cells, glycerol has several essential functions as it forms the backbone of the membrane forming phospholipids and also of triglycerides. Glycerol also acts as an organic osmolyte to maintain cell volume and integrity and as a chaperone to prevent protein aggregation. Glycerol is at the center of glucose and lipid metabolism in liver and other tissues. FFA, free fatty acids; Gro3P, glycerol-3-phosphate; TG, triglycerides; VLDL, very low-density lipoprotein. This figure was created using BioRender under license UJ28NXQ1WU to Elite Possik.

Glycerol is a 3-carbon, 3-hydroxyl-group aliphatic polyol with a molecular weight of 92.09. It is also called glycyl alcohol, glycerin, and 1,2,3-propanetriol. At room temperature, glycerol is a viscous, colorless, odorless, nontoxic liquid. It is widely used in the food, pharmaceutical, and cosmetics industries as a sweetener, preservative, thickening agent, humectant, osmolyte, and cryoprotectant. In the food industry, glycerol is used as an artificial sweetener because of its mild sweet taste, low glycemic index, humectant, and preservative properties (13, 14). Due to mild antimicrobial effects at high concentrations, and its water-retaining properties, glycerol is commonly used in hydrogel-type dressings for wound care (13, 15, 16). It is commonly used in topical applications for dry skin conditions and to enhance epidermal barrier function (9). Because of its osmotic effects, glycerol is used in suppositories and enemas to treat constipation (13). Also, as an organic osmolyte, glycerol is used to treat cerebral edema in traumatic brain injury (TBI) and other cerebral disease conditions (3); however, hypertonic saline or mannitol are now more commonly used in these circumstances (17). Glycerol has an established role in cryopreservation of various cells and tissues, including skin, corneas, sperm, and embryos (18-20). This relates to its antifreeze properties (preventing ice formation) and capacity to act as a chemical chaperone, which can help stabilize proteins, preventing aggregation (4, 21, 22). Some of the characteristics, properties, and cellular functions of glycerol are illustrated in Fig. 1.

## Overview of Glycerol in Whole-Body Metabolism

**Figure 2.**
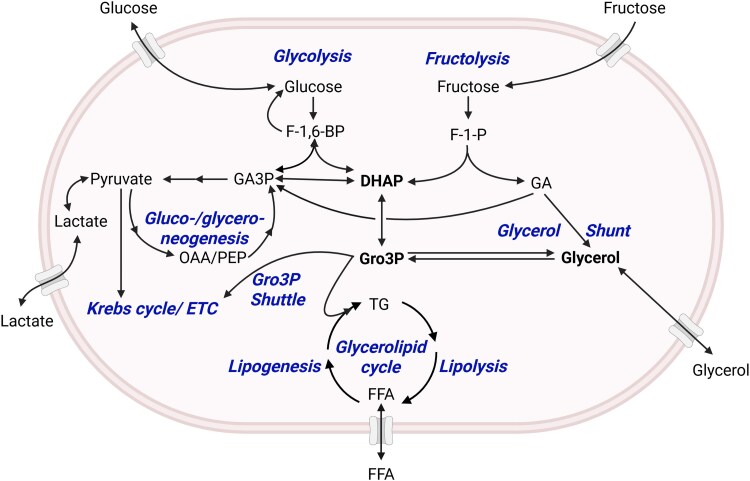
Glycerol metabolism in a nutshell. Glycerol metabolism constitutes a central part in the overall intermediary metabolism connecting multiple pathways. The catabolism of glucose via glycolysis and of fructose via fructolysis generates DHAP, which partitions among many pathways. DHAP can be reduced to Gro3P to enter the Gro3P electron shuttle for the transfer of cytosolic-reducing equivalents to mitochondria or serve as the backbone for TG synthesis via lipogenesis in the glycerolipid cycle. Pyruvate formed during glycolysis can be reduced to lactate and transported out of the cell or enter into the Krebs cycle for complete oxidation. Pyruvate formed from other sources can also be used for gluconeogenesis to generate glucose via OAA and PEP, followed by the reversal of glycolysis to form glucose. Pyruvate-derived GA3P can also give rise to Gro3P via DHAP, a process known as glyceroneogenesis. DHAP can also be metabolized in a newly identified pathway in mammalian cells named the glycerol shunt in which DHAP is reduced to Gro3P, which is then hydrolyzed to glycerol via Gro3P phosphatase. Fructolysis also generates GA, which can be phosphorylated by triose kinase to GA3P or reduced to glycerol by alcohol dehydrogenase. Glycerol formed from either mechanism can be transported out of the cell, thereby shunting the sugar carbons out of the cell. Blood glycerol can enter some cells to be phosphorylated by glycerol kinase to Gro3P, which then enters many pathways. DHAP, dihydroxyacetone-3-phosphate; ETC, electron transport chain; F-1-P, fructose-1-phosphate; F-1,6-BP, fructose-1,6-biphosphate; FFA, free fatty acids; GA, glyceraldehyde; GA3P, glyceraldehyde-3-phosphate; Gro3P, glycerol-3-phosphate; OAA, oxaloacetate; PEP, phosphoenolpyruvate; TG, triglycerides. This figure was created using BioRender under license BU28NLOH5D to Pegah Poursharifi.

Glycerol and glycerol-3-phosphate (Gro3P) have central roles in carbohydrate and lipid metabolism (Fig. 2). As the backbone of neutral and phosphoglycerolipids, glycerol has critical roles in energy storage mostly in the form of TGs, in cellular membranes as the phospholipid component, and in the generation of lipid-signaling molecules through the glycerolipid (GL) cycle (23). Glycerol and Gro3P have additional roles in energy metabolism within the gluconeogenesis pathway, the mitochondrial Gro3P shuttle, and in substrate supply for mitochondrial metabolism (24, 25). Gro3P can be produced by phosphorylation of free glycerol by glycerol kinase (GK), from lactate or alanine via glyceroneogenesis (25), as well as from the activities of the glycolysis and fructolysis pathways and from glycogenolysis (25, 26). Free glycerol can be produced from lipolysis of GLs (23), the reduction of glyceraldehyde in the fructolysis pathway (26), and importantly from hydrolysis of Gro3P by Gro3P phosphatase (G3PP) within the recently described glycerol shunt (6, 7). The activities of these pathways vary across organs and depend very much on nutritional state. We discuss the different aspects of glycerol metabolism in various tissues next.

### Liver, Kidneys, Skeletal Muscle, Heart, and White Adipose Tissue

**Figure 3.**
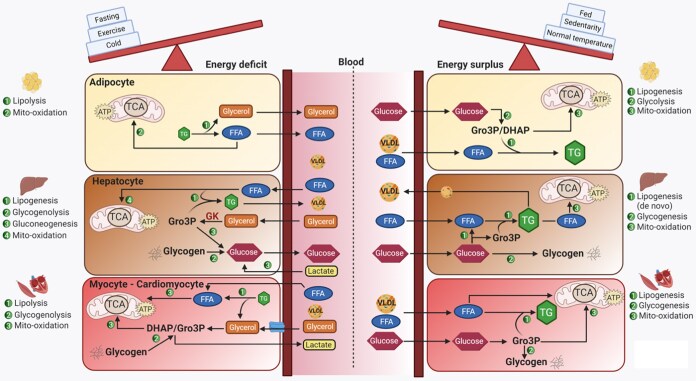
Glycerol metabolism and intertissue communication via blood glycerol and other fuel metabolites according to energy state. Metabolites that participate in glycerol metabolism are shared by different tissues through circulation. Here, we show examples of the main body organs adipose tissues, liver, and muscle. Under energy-deficit conditions like fasting, exercise, or cold stress, TG lipolysis in adipose is enhanced to produce glycerol and FFA, which are released into blood, to be used by other tissues like liver or muscle. Some of the FFA produced can also be oxidized in mitochondria in the adipocytes, with high oxidation in brown adipose tissue. Under energy-surplus conditions, such as feeding, sedentary state, or at ambient room temperature, lipogenesis is enhanced in various tissues, in particular in the adipocyte, as glucose and FFA are used to produce TG. Hepatocytes, under energy-deficit conditions, pick up glycerol from blood to form glucose via gluconeogenesis. Glucose is also released from hepatocyte glycogen stores under energy deficit and FFA are diverted more toward mitochondrial oxidation than for TG synthesis and VLDL production. On the other hand, when energy is abundant, hepatocytes use blood glucose to synthesize glycogen and FFA via de novo lipogenesis, store TG, and secrete VLDL. Relatively, smaller amounts of FFA are oxidized in hepatocytes under an energy-surplus state. In muscle cells, there is enhanced lipolysis under energy deficit, with elevated glycerol and FFA production, to be used for oxidation. Glycogenolysis is also elevated in the myocytes under a low-energy state, to supply the much needed glucose for energy production. In the muscle cells, under an energy-surplus state, there is increased availability of lipoproteins/FFA and glucose from circulation, for some energy production and also storage as TG and glycogen, with little to no glycerol production. DHAP, dihydroxyacetone-3-phosphate; FFA, free fatty acids; GK, glycerol kinase; Gro3P, glycerol-3-phosphate; TG, triglycerides; TCA, tricarboxylic acid cycle; VLDL, very low-density lipoprotein. This figure was created using BioRender under license OT28NXPKCH to Elite Possik.

During fasting, exercise, and in response to stress, the main source of glycerol is from lipolysis, mostly from adipose tissue stores, resulting in the release of glycerol and FFAs into the circulation (27) (Fig. 3). In most tissues, glycerol has to be first phosphorylated to Gro3P by GK to participate in any further metabolic pathway. GK is most highly expressed in the liver and present in the kidney, and glycerol derived from lipolysis is used by these tissues for gluconeogenesis (3, 28). The liver, however, accounts for approximately 80% of the glycerol utilization by its conversion to glucose (3, 28). In tissues such as the heart and skeletal muscle, Gro3P produced via GK can enter the lower glycolysis pathway and mitochondrial metabolism as an energy source for adenosine triphosphate (ATP) production (29). Gro3P can also be derived from lactate, pyruvate, and alanine via glyceroneogenesis, which is particularly important for the activity of GL cycling in white adipose tissue (WAT), which does not normally express GK (30).

The distribution of glycerol utilization between the liver, kidney, and other tissues depends not only on the GK expression levels in these organs (3, 28) but also on whether the supply of glycerol is through portal or systemic circulation, and on nutritional state (29). Thus, in overnight-fasted male individuals, orally administered glycerol, which enters portal circulation, was shown to be predominantly converted to glucose by the liver, whereas intravenously administered glycerol is mostly metabolized by peripheral tissues to lactate (29). The percentage contribution of glycerol to total glucose production in humans increases from 5% under healthy-fed conditions to 20% or more on extended fasting (25).

In the fed state, sources of glycerol include food, hydrolysis of TGs carried in chylomicrons and very low-density lipoproteins (VLDLs) by lipoprotein lipase, and the activity of the glycerol shunt (6, 25). In this situation, Gro3P is used for GL synthesis in many tissues, particularly in the liver, muscle, and adipose tissue. As WAT does not normally express GK, glycerol released by the action of lipoprotein lipase on circulating chylomicrons and VLDL cannot be used by this tissue. Thus, Gro3P for TG synthesis within WAT is sourced from glucose and the glycolysis pathway, and glyceroneogenesis (30). GK expression in WAT, however, can be increased by treatment with thiazolidinediones, which promote TG synthesis and intracellular recycling of glycerol via the GL cycle (31). High-fat diet (HFD) feeding of mice has also been shown to induce GK expression in WAT, which has been linked to increased fat accumulation (32). How glycerol accumulation causes an induction of GK gene expression is not known, and a similar phenomenon has been reported in pancreatic β cells (33). It would be interesting to examine if glycerol has any transcriptional modulatory effects. Interestingly, it has been reported recently that the glycerol metabolite Gro3P directly binds to the transcription factor carbohydrate response element binding protein (ChREBP), and likely promotes its activity and the transcription of fibroblast growth factor-21 (FGF21) and lipogenesis in mouse hepatocytes (34).

### Brown Adipose Tissue

In rodents, brown adipose tissue (BAT), as opposed to WAT, expresses high levels of GK and this expression increases on cold exposure and in response to peroxisome proliferator-activated receptor γ (PPARγ) activation (35). BAT can recycle the intracellular brown adipocyte lipolysis-derived glycerol for reesterification via the formation of Gro3P by GK, and this action of GK is important for the thermogenic function of BAT (35).

### Intestine

Within the gut, pancreatic lipase predominantly hydrolyses dietary TGs to monoglycerides (MAGs) and fatty acids, with reesterification in enterocytes via the MAG acyltransferase pathway and chylomicron synthesis (36). Only about 30% of TGs are completely hydrolyzed in the intestine, and the released glycerol rapidly enters enterocytes of the intestinal wall and then into the blood via the portal vein, to be further metabolized mostly in the liver (37). Initially, it was believed that glycerol from intestinal sources (in foods or from fat digestion) could not be used for reesterification and regeneration of TGs in the intestinal enterocytes (38). However, GL synthesis from Gro3P is also present in enterocytes, but is less active than from MAG (36). Later studies revealed the presence of GK in the intestinal mucosa and utilization of glycerol for enterocyte GL synthesis (39). Moreover, it was observed that both fructose and glucose are capable of supplying the glycerol backbone for TG synthesis in enterocytes (40). In addition, the intestine, which normally conducts gluconeogenesis using glutamine (41), can also use glycerol as a substrate for gluconeogenesis when hepatic gluconeogenesis is compromised (28).

## Key Enzymes and Pathways in Glycerol and Glycerol-3-Phosphate Metabolism

**Figure 4.**
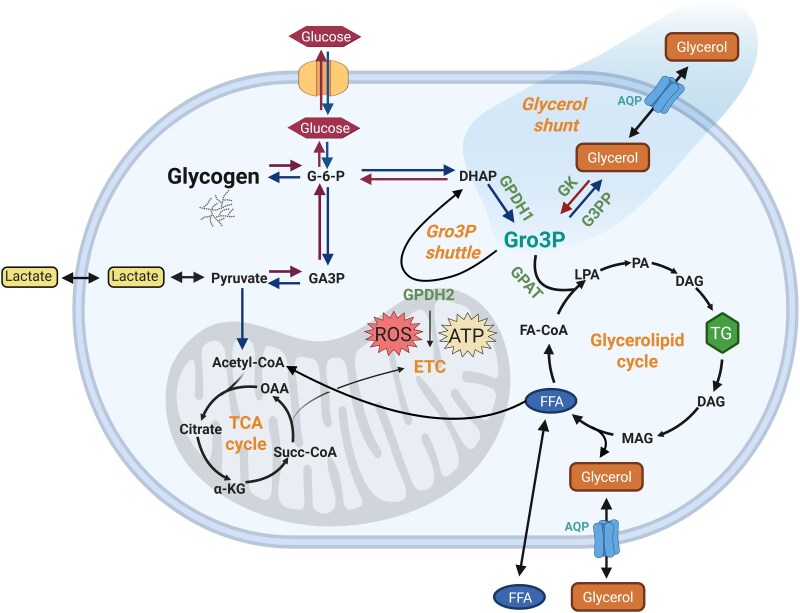
Glycerol metabolism and the glycerol shunt as illustrated in hepatocytes. Metabolic pathways active when calorigenic nutrients are abundant or during energy deficit are indicated by the lighter and darker arrows, respectively. Gro3P is at the intersection of carbohydrate and lipid metabolism as it can be used by the Gro3P shuttle for electron transfer to the mitochondria and the synthesis of ATP; it can be metabolized in lower glycolysis and can be used for TG synthesis via the glycerolipid cycle. This cycle constitutes sequential addition of FFA to the glycerol backbone of Gro3P to form TG followed by lipolysis, the stepwise hydrolysis of TG to glycerol and FFA. Current textbooks of biochemistry do not mention an important additional pathway of glycerol metabolism, the glycerol shunt (DHAP to Gro3P to glycerol), which has been identified recently in mammalian cells following the discovery of an ubiquitous enzyme (G3PP) that can directly hydrolyze Gro3P to glycerol. This pathway bypasses the glycerolipid cycle to produce glycerol and shunts the glucose carbons of glycolysis to glycerol. Glycerol leaves the cell through AQP. Glycerol can also enter the cell and in conditions of energy deficit be phosphorylated by GK to Gro3P and used either for gluconeogenesis or other pathways. AA, amino acids; AQP, aquaglyceroporin; DAG, diacylglycerol; DHAP, dihydroxyacetone-3-phosphate; ETC, electron transport chain; FA-CoA, fatty acyl-CoA; FFA, free fatty acids; G-6-P, glucose-6-phosphate; GA3P, glyceraldehyde-3-phosphate; GK, glycerol kinase; GPDH1 and 2, Gro3P dehydrogenase 1 and 2; Gro3P, glycerol-3-phosphate; G3PP, Gro3P phosphatase; α-KG, α-ketoglutarate; LPA, lysophosphatidic acid; MAG, monoacylglycerol; OAA, oxaloacetate; PA, phosphatidic acid; ROS, reactive oxygen species; Succ-CoA, succinyl-CoA; TG, triglycerides. This figure was created using BioRender under license FQ28NXPUKU to Elite Possik.

In this section, we discuss key enzymes and pathways involved in glycerol and Gro3P metabolism, including the effect of several enzyme mutations within them (Figs. 2 and  4). Well-known pathways such as glycolysis and gluconeogenesis are not discussed.

### Glycerol Kinase

**Figure 5.**
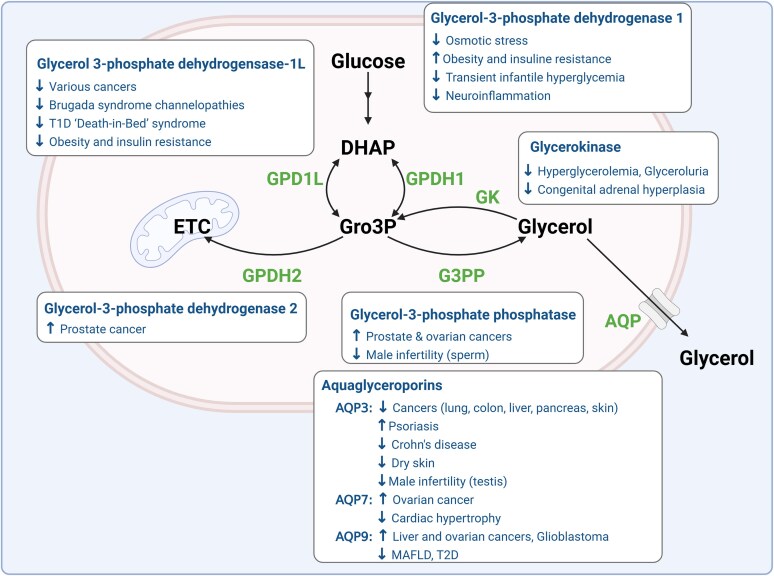
Pathological implications of disturbances in glycerol metabolism. In several pathological conditions, glycerol metabolism and the glycerol shunt pathway are dysregulated resulting in altered levels of plasma, urine, and tissue glycerol levels. Moreover, genetic deficiencies or defective function of the components of the glycerol metabolism or the glycerol shunt are associated with various diseases. Thus, defects in glycerol-3-phosphate dehydrogenase-1L (GPD1L) are found in various cancers, channelopathies like Brugada syndrome, obesity, and insulin resistance, etc. While decreased activity of glycerol-3-phosphate dehydrogenase-1 (GPDH1) is seen in osmotic stress conditions, neuroinflammation, and transient infantile hypertriglyceridemia, its activity is elevated in obesity and insulin resistance. Elevated activities of GPDH2 and also glycerol-3-phosphate phosphatase (G3PP) have been reported in prostate cancer whereas decreased activity of G3PP is associated with male infertility. Genetic deficiency of glycerol kinase (GK) leads to elevated blood and urinary glycerol levels and also congenital adrenal hyperplasia. Increased activity/expression of AQP3, 7, and 9 are associated with different cancers. On the other hand, decreased expression of AQP3 is seen in conditions of dry skin, Crohn disease, and male infertility, and lower AQP7 level in cardiac hypertrophy, and reduced expression of AQP9 in MAFLD and T2D. AQP, aquaglyceroporin; DHAP, dihydroxyacetone-3-phosphate; ETC, electron transport chain; G3PP, Gro3P phosphatase; GK, glycerokinase; GPD1L, Gro3P dehydrogenase-1 like; GPDH1, Gro3P dehydrogenase-1; Gro3P phosphatase; MAFLD, metabolic dysfunction–associated fatty liver disease; T1D, type 1 diabetes; T2D, type 2 diabetes. This figure was created using BioRender under license LS28NME4GE to Pegah Poursharifi.

GK, which phosphorylates glycerol to Gro3P, is encoded by the *GK* gene comprised of 21 exons that map to chromosome Xp21.3. As discussed previously, GK is an essential entry point for glycerol into the metabolism, with high activity in the liver. Mutations and deletions of loci on chromosome Xp21 have been linked to GK deficiency syndrome, characterized by hyperglycerolemia, glyceroluria, congenital adrenal hypoplasia, and developmental delay (42-48) (Fig. 5). *GK* in mature BAT is a PPARγ-regulated gene, proposed to be important in PPARγ's role in driving the thermogenic capacity of this tissue, including its response to β-adrenergic signaling (35). GK expression has also been shown to be induced by several fold in BAT by cold exposure via sympathetic regulation (49). In the heart, islet β cells, and adipose tissue, evidence from AQP7-knockout (KO) mice suggests that *GK* gene expression is directly related to cellular glycerol content (33, 50, 51). GK, as well as hexokinase, interact with porin, a voltage-dependent anion channel on the mitochondrial outer membrane, which may be important for micro-compartmentation of the enzyme activity close to mitochondria (52). In addition to its function as a phosphotransferase, GK has been shown to act as an ATP-stimulated translocation promoter that facilitates nuclear binding of the glucocorticoid-receptor complex (53, 54).

### Glycerolipid Cycle

This cycle is central to the synthesis of GLs and associated signaling molecules (Fig. 4). It initiates with lipogenesis that involves the sequential esterification of the glycerol backbone of Gro3P, forming lysophosphatidic acid (LPA), phosphatidic acid, *sn*1,2-diacylglycerol, and finally TGs. Once formed, TGs are stored as lipid droplets, which are dynamic with continuous turnover, via lipolysis and reesterification of FFAs. Lipolysis, that is, the hydrolysis of GLs starting from TGs, sequentially generates *sn*1,3- and *sn*2,3-diacylglycerols, 1- and 2-monoacylglycerols, and finally glycerol and FFAs, which either leave the cell or reenter the GL cycle (23, 55). Several of the lipid intermediates produced during the course of lipogenesis and lipolysis have signaling properties depending on their site and context of production (23, 55, 56). Not all the Gro3P that enters the GL cycle is derived from glycerol phosphorylation, as GK expression is restricted to few tissues (eg, liver and BAT), and the Gro3P supply for the GL cycle in those tissues with low GK can be provided via glyceroneogenesis or the reduction of dihydroxyacetone phosphate (DHAP) formed during glycolysis (23). The lipolysis arm of the GL cycle produces free glycerol, which in cells without GK leaves the cell through AQP channels.

### Glyceroneogenesis

Gro3P in cells can also be synthesized from sources other than glucose, in particular lactate, pyruvate, and alanine, a pathway initially described and termed “*glyceroneogenesis*” (see Fig. 2) (57, 58). It was first shown that rat epididymal fat pads can incorporate pyruvate into the glyceride glycerol under conditions of glucose deprivation (57). Glyceroneogenesis involves the conversion of pyruvate, arising from lactate, alanine, or other amino acids, to oxaloacetate by mitochondrial pyruvate carboxylase, followed by the action of cytosolic phosphoenolpyruvate (PEP) carboxykinase-GTP (PEPCK) to produce PEP, which subsequently goes through a reversal of a part of glycolysis to form DHAP and then Gro3P. Glyceroneogenesis is of particular importance for the provision of Gro3P to support GL synthesis and the GL cycle under fasting and diabetic conditions. Glycerol from adipose tissue lipolysis during fasting cannot be used for FFA reesterification in WAT due to low levels of GK, and very little of this lipolysis-generated glycerol is used (∼3%) for TG synthesis in the liver, despite high activity of GK in the liver (59, 60). Therefore, an alternate supply of Gro3P is required for GL cycling in WAT and GL synthesis in the liver under fasting and diabetic conditions, which comes from glyceroneogenesis, dependent on the activity of PEPCK (23, 58). Glyceroneogenesis contributes to more than 65% of the glyceride glycerol produced in liver under fasted conditions, whereas only approximately 3% of the blood glycerol is used for TG synthesis via GK-mediated Gro3P formation (59, 60). This suggests that in hepatocytes, Gro3P formed from plasma glycerol is channeled mostly for gluconeogenesis, whereas glyceroneogenesis supplies Gro3P for TG synthesis, even though the underlying mechanisms for such substrate compartmentalization are not clear (60). Glyceroneogenesis, as a source of Gro3P, has also been shown to be important for nonshivering thermogenesis via ATP turnover from GL cycling in BAT (30, 60, 61). This elevated Gro3P supply via glyceroneogenesis is due to the increased activity of PEPCK in BAT in response to cold exposure in mice (62). Glyceroneogenesis could also have a role in intestinal epithelial cell TG synthesis, as the intestine also expresses PEPCK (63).

### Glycerol-3-Phosphate Shuttle

Different mechanisms, including the Gro3P shuttle, exist to transfer the electrons from cytosolic NADH to the mitochondrial electron transport chain for the production of ATP via oxidative phosphorylation. The Gro3P shuttle consists of two enzymes, glycerol-3-phosphate dehydrogenase 1 (GPDH1) in the cytosol and GPDH2, a flavoprotein, bound to the cytosolic surface of the inner mitochondrial membrane (see Fig. 4). The transfer of reducing equivalents from cytosol to mitochondria is accomplished in two steps. First, by GPDH1-mediated reduction of DHAP to Gro3P, using NADH, followed by GPDH2-mediated oxidation of Gro3P back to DHAP on the mitochondrial surface, transferring electrons from Gro3P to FAD to produce FADH2. FADH2 then enters the mitochondrial electron transport chain via coenzyme Q at complex II for ATP production (24). GPDH2 is a major site of reactive oxygen species (ROS) production in mitochondria (64). This shuttle uses glucose-derived Gro3P, though glycerol itself can enter the Gro3P shuttle after its phosphorylation to Gro3P, depending on the expression level of GK in the cells. GPDH2 expression is likely regulated by cellular glycerol, as noticed in AQP7-KO mouse cardiac tissue, in which lowered glycerol uptake and content of heart tissue is associated with reduced GPDH2 expression (50). Morbidly obese people were found to display elevated GPDH1 activity, suggesting a pro-obesity role for this enzyme, whereas loss of GPDH1 function was found to cause transient infantile hypertriglyceridemia and also neuroinflammation (65).

### Glycerol-3-Phosphate Dehydrogenase 1-Like

Gro3P dehydrogenase 1-like (GPD1L) is a cytosol-facing, plasma membrane-bound enzyme that has 84% homology with GPDH1 and catalyzes the same reaction as GPDH1. *GPD1L* is present on chromosome 3p22.2 and is highly expressed in the heart, but minimally expressed in skeletal muscle and other tissues (66, 67). The relative contribution of this enzyme to the overall DHAP to Gro3P flux is not known. Loss-of-function mutations in this enzyme are associated with multiple diseases (see Fig. 5), including cancers (68), channelopathies like Brugada syndrome (67, 69, 70), and type 1 diabetic “death-in-bed” syndrome (71). Further, downregulation of *GPD1L* is linked to obesity and insulin resistance (72). Whether some of these associations are causal in these diseases remains to be shown.

### Glycerol-3-Phosphate Phosphatase and the Glycerol Shunt

Until our textbook discovery that mammalian cells harbor G3PP that hydrolyzes Gro3P (6), it was believed that the fate of Gro3P was either via the GL synthesis pathway or the Gro3P shuttle. We have termed this Gro3P hydrolysis pathway the “*glycerol shunt*” and, considering its recent discovery and key roles within many pathways of intermediary metabolism, as illustrated in Figs. 2 and 4, this enzyme and pathway are discussed next in more detail.

We showed that phosphoglycolate phosphatase (gene: *PGP*), a previously described ubiquitous enzyme with an uncertain primary function, actually hydrolyzes Gro3P to glycerol in response to an intracellular rise in Gro3P, so that the *PGP* gene product is now recognized by Swiss-Prot as G3PP (6, 7). The production of glycerol directly from glucose-derived Gro3P was also suggested in the rat brain and 3T3-L1 adipocytes, even though the enzyme involved was not identified (73, 74). G3PP/PGP was also described as aspartate-based, ubiquitous, Mg^2+^-dependent phosphatase, with weak phosphotyrosine phosphatase activity, though its intracellular relevance was not demonstrated (75). A few studies indicated that G3PP/PGP can also hydrolyze the toxic glycolytic reaction side products 4-phosphoerythronate and 2-phospho-L-lactate in cancer cells (76), emphasizing the importance of this enzyme in metabolic stress conditions. However, under normal physiological conditions in mammalian cells, phosphoglycolate, 4-phosphoerythronate, 2-phospho-L-lactate are at negligible levels, whereas intracellular Gro3P concentrations are at near or above the Km for G3PP/PGP (6, 7), supporting the view that G3PP's primary function is the hydrolysis of Gro3P.

G3PP is evolutionarily conserved from yeast and invertebrates to vertebrates and mammals. In yeast, two isoforms of the Gro3P phosphatases exist: Gpp1p and Gpp2p (77). In *C elegans*, we have recently identified 3 isoforms and named them *pgph-1*, *pgph-2*, and *pgph-3* (78). In humans, *PGP* consists of 2 exons and 1 intron spanning approximately 4 Kbp of the human genome and is mapped to chromosome 16 at the 16p13.3 loci (79-81), while in the mouse it is on chromosome 17. G3PP is ubiquitously expressed with high expression in testis, cardiac and skeletal muscles, liver, and pancreatic islets, as shown in the Human Protein Atlas database (82).

In mice, global inactivation of *PGP* (*PGP*^D34N/D34N^) is embryonically lethal due to intrauterine growth arrest in midgestation, and in *PGP*^D34N/D34N^ mouse embryonic fibroblasts cell proliferation is impaired and GL metabolism is dysfunctional with higher TGs, but lower phosphatidylcholine content, indicative of the importance of G3PP in development and GL metabolism (83). Inducible tissue-specific deletion of PGP/G3PP in hepatocytes and the pancreatic β cell showed no evidence of tissue or cell toxicity, unless stressed by elevated glucose in vivo or ex vivo (84, 85). Hepatocyte-specific G3PP-KO mice showed liver inflammation and steatosis when hyperglycemia was induced by a 55-hour glucose infusion (84). G3PP-deficient hepatocytes cultured in high glucose concentrations had increased lactate and TG production and reduced glycerol production (84). Inducible tissue-specific deletion of G3PP in pancreatic β cells of mice fed a normal chow diet showed increased mouse body weight, adiposity, fed-state insulinemia, enhanced in vivo and in vitro glucose-stimulated insulin secretion, reduced plasma TGs, and mild glucose intolerance (85). Under only short-term high-glucose conditions, G3PP-deficient islets were shown to have reduced glycerol release, increased O_2_ consumption and ATP production, as well as increased insulin secretion. Under chronic (7 days’) exposure to elevated glucose, islets from the KO mice showed higher rates of apoptosis (85). By contrast, overexpressing the enzyme in INS832/13 β cells, primary hepatocytes, and liver in vivo had opposite effects (6).

The glycerol shunt pathway (DHAP to Gro3P to glycerol), for which G3PP is key, is termed as such, as it is able to shunt glucose-derived carbons to glycerol, away from other pathways of glucose and Gro3P metabolism (see Fig. 4). The glycerol shunt is particularly active at elevated glucose concentrations and was found to be directly related to the expression level of G3PP in different cell types and tissues, including INS-1(832/13) rat β cells, rat and mouse islets, rat and mouse primary hepatocytes, and in *C elegans* (6, 78, 84, 85). Considering that the Gro3P level in the cell increases in the presence of high concentrations of glucose, we postulated that G3PP is a nutritional stress-response enzyme, which was confirmed in our studies using mammalian cells (84, 85) as well as in a *C elegans* model (11, 78). Furthermore, a recent study showed that various types of cancer cells, when acutely (8 hours) exposed to hypoxic conditions (0.5% O_2_) such as those existing in solid tumors, produce markedly elevated levels of glycerol, a process dependent on the activity of G3PP/PGP (86). Such increased glycerol production was not seen under normoxic conditions, again suggesting that G3PP is a stress-related enzyme that alleviates reductive stress in the cancer cells under hypoxia (86).

Overall, it appears that G3PP and the glycerol shunt protect cells from metabolic stress, act as a “glucose excess detoxification machine” or “reductive stress security valve” and through this feature have a substantial influence on glucose, lipid, and energy metabolism, particularly at high glucose.

### Fructolysis

High consumption of fructose (∼9%-25% of total caloric intake in North America) is associated with metabolic diseases including metabolic dysfunction–associated steatotic liver disease (MASLD), type 2 diabetes (T2D), and even certain cancers (87-89). Plasma fructose concentrations under fasting conditions are approximately 0.03 mM, and this can increase many fold following a high-fructose meal or soft drink (up to 0.3-1.5 mM) (88, 90). It was reported that patients with pancreatic cancer had high levels of plasma fructose vs healthy controls (5.7 vs 1.9 mM) (91). Through fructolysis, fructose can be an additional source of glycerol, which may feed into TG synthesis and contribute to its detrimental effects on metabolic health.

Fructolysis (see Fig. 2) begins with the formation of fructose-1-phosphate by ketohexokinase. Aldolase B then converts fructose-1-phosphate into DHAP and glyceraldehyde, and as fructolysis does not involve the rate-limiting and regulatory enzyme phosphofructokinase, metabolism of fructose is not controlled by the same feedback mechanisms that operate for glycolysis. Glyceraldehyde is a branch point in fructolysis as it can be converted by triose kinase to glyceraldehyde-3-phosphate (GA3P) to enter lower glycolysis, or by aldehyde dehydrogenase to glycerate to be used for serine biosynthesis or further oxidation, or alcohol dehydrogenase to glycerol (26). Indeed, depending on the consumption of fructose, a significant amount of glycerol can be formed from this sugar (see Fig. 2). GA3P, glycerol, or DHAP can be readily converted to Gro3P and used for GL synthesis (26).

Because of the restricted distribution of fructose transporters (GLUT5 in the intestine and GLUT2 in the liver) and the enzymes involved in its metabolism, fructose is mostly metabolized in the intestine or in the liver (88, 92). Indeed, it has been shown recently that a significant part of ingested fructose is metabolized in the intestine as intestine-specific deletion of ketohexokinase, which catalyzes the first step of fructose metabolism, leads to elevated fructose levels in the portal circulation (87, 93). Interestingly, adipocytes, which do not have aldolase B, can still divert a significant portion of fructose toward glycerol formation, suggesting the presence of an alternate pathway (74).

Considering the strong associations of excess fructose with MASLD (87-89) and that fructose with glucose can cause tissue toxicity via glycation processes (94, 95), it will be of interest to examine if the glycerol shunt also plays a role in the detoxification of excess fructose (see Fig. 2), particularly in the liver where this sugar is highly metabolized.

## Aquaglyceroporins and Transmembrane Transport of Glycerol

Glycerol is transported across cell membranes by passive transport via aquaglyceroporins, which belong to the family of pore-forming transmembrane proteins called AQPs, which facilitate the diffusion of water and certain other small molecules across the plasma membrane. Structurally, all AQPs have 6 transmembrane domains and share highly conserved asparagine-proline-alanine motifs, on both the C-terminal and N-terminal half of the protein, which face the cytosolic side (96). Monomeric (∼30 kDa) AQPs form tetramers in membranes, resulting in a tetrameric pore (97). Thirteen distinct isoforms are specific to humans and distributed across different tissues (5). AQP3, AQP7, AQP9, and AQP10 can transport glycerol in addition to water, and other small uncharged molecules such as urea, and are called aquaglyceroporins. Altered expression of AQP3, -7, and -9 are associated with different pathological conditions as illustrated in Fig. 5.

### Aquaglyceroporin 3

AQP3 is expressed in epithelial cells of several tissues, with roles particularly understood in skin, kidneys, and intestine. AQP3 is abundantly expressed in strata basale and spinosum of the epidermis, where, via the humectant properties of glycerol, it contributes to hydration of the stratum corneum, skin elasticity, barrier function, and wound healing (98-101). Oral glycerol replacement was able to correct defective skin phenotypes in AQP3 null mice (102). In the kidney, AQP3 is located in the basolateral membrane of collecting duct epithelial cells, and its deletion in mice causes nephrogenic diabetes insipidus (103, 104) and can exacerbate renal ischemia-reperfusion injury (104). In the terminal ileum of patients with Crohn disease, AQP3 expression both at the messenger (mRNA) and protein level is reduced in mucosal epithelial cells, which may reflect loss of the normal cell polarity as a consequence of inflammation and contribute to diarrhea by reducing intestinal water absorption (105). AQP3 is also expressed in various parts of the brain (106-108) and may play a role in brain edema and neuronal swelling following brain ischemia (109).

### Aquaglyceroporin 7

AQP7 is expressed in many tissues, including adipose, endocrine pancreas, testis, digestive tract, eye, kidney, muscle, heart, as well as in the microvasculature of some tissues. Its established roles are in metabolism, including adipocyte TG storage and release, glucose homeostasis, insulin secretion, and body weight (37, 51, 110-114), as well as renal tubular glycerol reabsorption (115), and male fertility (116). Structural analysis of AQP7 suggests that, in the presence of glycerol, this transporter favors transport of glycerol over H_2_O (117).

AQP7 has a key role in glycerol efflux from adipocytes at times of lipolysis activation (eg, during fasting and exercise), through translocation to the plasma membrane from being intracellularly bound to perilipin-1 associated with lipid droplets, a result of phosphorylation by hormonally regulated protein kinase A (118, 119). This role of AQP7 in adipocyte lipid metabolism is evident in AQP7-KO mice, in which WAT lipid droplet size is increased, intracellular glycerol content is increased, and GK activity (usually absent in adipocytes) is elevated, along with increased fat mass, body weight, and insulin resistance (33, 51, 111, 112). Prolonged fasting in the AQP7-KO mouse results in profound hypoglycemia, indicative of the importance of adipose glycerol efflux for liver gluconeogenesis (111). In a human with a missense mutation of AQP7, the normal increase in glycerol in response to exercise did not occur, further evidence of the importance of AQP7 in adipose glycerol efflux (120, 121). Of note, AQP7 expression in adipose tissues is dysregulated in obese and T2D patients (122-124).

Glycerol efflux in pancreatic islet β cells is also via AQP7, so that in AQP7-KO mice intracellular glycerol concentrations increase 2-fold, GK mRNA increases 7-fold, intraislet TG content rises, and despite lower islet β-cell mass, insulin secretion is increased (33). However, in cardiomyocytes of AQP7-KO mice, glycerol uptake is lowered, and this is associated with reduced expression of GK and mitochondrial Gro3P dehydrogenase-2 (50). AQP7-KO mice are prone to develop cardiac hypertrophy and have higher mortality in response to cardiac stressors, indicative of the importance of glycerol to cardiomyocyte function (50). Due to the association of intracellular glycerol content with the expression of GK in at least 3 cell types (adipocyte, islet β-cell, cardiomyocyte), it appears that intracellular glycerol can influence the expression of genes related to its own metabolism.

AQP7 is expressed in the apical membrane of the straight region of the proximal tubule of the kidney and, as shown from studies of AQP7-KO mice, is involved in glycerol reabsorption (115, 125, 126). Consistent with this renal role of AQP7, hyperglyceroluria has been documented in humans with homozygous AQP7 mutations (127). In the same individuals, AQP7 deficiency was associated with mild platelet dysfunction (127).

The ontogeny and distribution of AQP7 in rat testis suggested a role in spermatogenesis (116). In men, a lack in AQP7 expression is associated with infertility and the AQP7-deficient sperm show significantly reduced motility (128-130). However, male AQP7-KO mice are not sterile and have no histological abnormalities in their testes and sperm (113, 131).

### Aquaglyceroporin 9

AQP9, first identified in human leukocytes (132) and rat liver (133), is expressed in several other tissues, including skin, brain, and male and female reproductive tracts in humans, and conducts the transport of not only water and glycerol but also other small molecules such as urea, purines, and pyrimidines (132-135). AQP9 is the most highly expressed AQP in immune cells, with substantial evidence of a role in activated neutrophils (134). In the liver, AQP9 is mostly expressed in hepatocytes (133) and its expression is upregulated by starvation and lowered by insulin (136-138). Thus, AQP9 liver expression was shown to increase in rats with streptozotocin-induced type 1 diabetes and decrease following insulin injections (137). In clinically obese patients with insulin resistance and MASLD, the increased plasma glycerol levels are found to be associated with decreased expression of AQP9 in hepatocytes (139, 140). AQP9-KO mice, unlike AQP7-KO mice, develop elevated plasma glycerol levels, but do not become hypoglycemic when starved, as in the case of AQP7-KO mice, indicating compensation by other AQP or pathways and organs (37). The role of AQP9 in the brain both in health and in response to brain injury is less well understood (135, 141).

### Aquaglyceroporin 10

AQP10 is expressed in adipocytes in humans, whereas in mice and other species, it exists only as a nonfunctional pseudogene (142, 143). In vitro studies showed that AQP10 deletion in human adipocytes reduces water and glycerol permeability, supporting the view that AQP10 provides an alternative glycerol transport mode in addition to AQP7 in adipocytes (144). AQP10 is uniquely stimulated under the conditions of elevated lipolysis, when cytosolic pH becomes acidic, due to the protonation of a histidine residue on the cytosolic side of the AQP10 (145). AQP10 is also expressed in the gastrointestinal tract, skin, and male reproductive system, but its physiological significance is not clear.

## Glycerol in Blood, Urine, and Tissues

Intuitively one would believe that extracellular and intracellular glycerol concentrations would be in the same range as this osmolyte is thought to be transported by facilitated diffusion across cells by AQPs, and many AQPs are bidirectional, at least under in vitro conditions. However, measured concentrations of glycerol in tissues are surprisingly 3- to 300-fold higher than in plasma depending on tissue type, suggesting that intracellular concentrations are much higher. This raises questions as to whether the high glycerol concentrations measured in tissues accurately reflect in vivo concentrations, and if so, what is the intracellular distribution of glycerol (eg, free and bound to macromolecules, and within organelles) and its role.

### Glycerol Concentrations and Turnover in Blood

**Figure 6.**
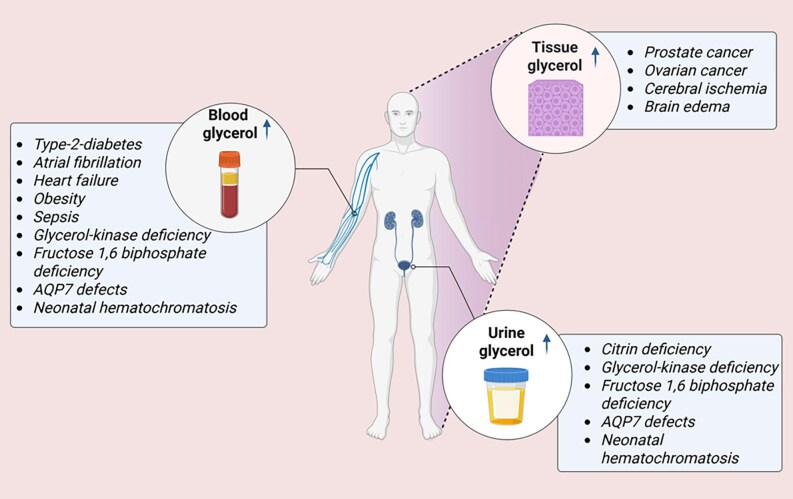
Elevated glycerol levels in tissues, plasma, and urine in various disease conditions. In humans, plasma glycerol concentration ranges widely from 0.01 to 0.5 mM, with a mean fasting concentration of 0.075 mM. In various disease conditions such as type 2 diabetes, obesity, etc, plasma glycerol levels increase to different extents. Genetic deficiencies of GK, AQP7, or citrin are associated with elevated urinary excretion of glycerol. Increased plasma glycerol levels are seen in T2D and are associated with atrial fibrillation, heart failure, and cardiovascular diseases and cancer mortality. Human tissue levels of glycerol are also found to increase, particularly in different cancer tissues and in brain edema and ischemia. The renal glycerol threshold is reached at a plasma glycerol concentration of approximately 0.33 mM, resulting in much less glycerol urinary excretion in normal healthy individuals. However, significantly elevated urinary excretion of glycerol is noted under conditions of citrin deficiency, glycerol kinase deficiency, etc. AQP, aquaporin. This figure was created using BioRender under license MQ28NXQWSU to Elite Possik.

Plasma glycerol concentrations in humans vary considerably from 0.01 to 0.5 mM, with a geometric mean fasting concentration of 0.075 mM measured in a cohort of 1056 individuals (3, 146), and it was suggested that despite the effect of environmental factors on glycerolemia, there is a high heritability (58%) of fasting glycerolemia (146). Women have higher plasma glycerol levels than men (3). In situations where lipolysis is activated, plasma glycerol concentrations increase, so that fasted concentrations are approximately 2-fold higher than in the fed state (147), and concentrations increase with exercise according to its intensity (28, 147, 148). In individuals with diabetes, fasting-state blood glycerol concentration is approximately 2-fold higher than that in nondiabetic individuals (146, 149), and plasma glycerol increases in diabetic ketoacidosis (DKA) (150). It was reported that blood glycerol levels reach approximately 0.4 mM in the first 48 hours after birth in newborn infants and thereafter slowly decline to approximately 0.2 mM by age 1 to 6 months (151). X-linked mutations in GK are associated with hyperglycerolemia, with plasma concentrations in affected individuals typically being greater than 2 mM (146) (Fig. 6).

While plasma glycerol concentrations in healthy fasted individuals are only approximately 1.5% of plasma glucose concentrations (0.075 mM compared to 4.7 mM), plasma glycerol turnover is approximately 21% that of glucose (3 vs 14 μmol/kg/min), and plasma glycerol clearance is approximately 14-fold higher than that of glucose (41 vs 3.0 mL/kg/min), indicative of substantial glycerol flux through the circulation despite low plasma levels (152). Thus, small variations in plasma glycerol concentration are likely to be associated with very significant changes in glycerol metabolism. This high glycerol flux through plasma in humans is in keeping with its estimated contribution to whole-body oxidative energy metabolism of about 10%, measured in terms of respiratory CO_2_ production (153). Glycerol turnover increases with exercise and during later stages of pregnancy, consistent with increased lipolysis (154, 155).

A recent study noticed that glycerol is the only nutrient in circulation that is poorly engaged in total energy expenditure in mice, compared to other nutrients in the blood (156). Flux analysis of glucose-derived metabolites in circulation both in ob/ob and HFD-fed obese mice vs control mice showed a markedly increased production only of glycerol and no change in other metabolites. This plasma glycerol could have been derived directly from glucose via the glycerol shunt as proposed by our group (84, 85, 157) and/or via VLDL-TG lipolysis. Overall, glycerol displays unique metabolism that is yet to be better understood (156).

### Glycerol in Urine

The maximal tubular resorptive capacity of the kidney is reached at a plasma glycerol concentration of approximately 0.33 mM, the renal glycerol threshold, so that in normal healthy individuals glyceroluria does not occur (28, 158). As already discussed, glycerol reabsorption from urine is predominantly via AQP7, so that homozygous mutations of AQP7, or proximal tubule dysgenesis as occurs in neonatal hemochromatosis, can cause glyceroluria (127, 159). Circumstances in which plasma glycerol levels increase above the renal threshold, as in cases of mutated GK (42-48), or with oral or intravenous glycerol supplementation (158), cause glyceroluria. A deficiency of the mitochondrial aspartate/glutamate carrier-2 (AGC2), also known as citrin deficiency, was found to be associated glyceroluria, although the mechanism is not understood (160) (see Fig. 6).

### Glycerol Levels in Various Cell Types and Tissues

A search of published data on glycerol concentrations in animal tissues and cells yielded numbers that vary considerably depending on cell type and the physiological or pathological status (see Fig. 6). The methodology described is not always clear, and it is possible that lipolysis of GLs during sample preparation may have artificially elevated the concentrations. Furthermore, the methods adapted for glycerol measurements in most of these studies employed commercially available kits, which measure glycerol after its conversion to Gro3P. Using such a kit-based method also measures Gro3P in the tissue/cell extracts as no corrections are made for endogenous Gro3P levels. Thus, many of the published values of glycerol levels in tissues and also in blood/plasma, described next, likely include not only glycerol but also Gro3P.

#### Brain

Glycerol content in brain tissue has been measured, prior to cerebral injury via trauma or ischemia in rats, cats, and gerbils, with levels of 6.5 nmol/mg (intracellular), 0.29 nmol/mg, and 0.19 nmol/mg, respectively, being reported (161-163). Assuming brain water content of approximately 76% (164), these contents per tissue weight can be converted to, respectively, 8.6 mM, 0.38 mM, and 0.25 mM, which is approximately 3.3 to 86 times higher than plasma glycerol (assuming a plasma concentration of ∼0.075 mM). The measured brain glycerol contents increased by approximately 80% 48 hours after TBI in rats, and by approximately 3.6-fold and approximately 9-fold after 15 minutes of brain ischemia in cats and gerbils, respectively, most likely due to activation of lipolysis secondary to the injury, but also as a consequence of upregulation of AQP9 (161-163).

#### Islets

In the pancreatic islets of normal 4-hour fasted mice, glycerol content was reported to be approximately 12 µg/mg protein (∼51 mM) in male and approximately 17 µg/mg protein (∼72 mM) in female mice (33). Islet glycerol content of AQP7-KO mice was about 2-fold higher (33).

#### Adipocytes

Female mouse gonadal adipocyte glycerol content has been reported to be approximately 80 nmol/mg protein (112). Assuming that 1 mg of protein is distributed in a 2.56-µL fat droplet free intracellular volume (based on hepatocyte protein per cell volume analyses (165)), the adipocyte glycerol concentration can be calculated to be approximately 31 mM.

#### Skin

In mouse skin, glycerol content in epidermis was reported as 37 nmol/mg protein (100), which translates to approximately 14.4 mM glycerol, again assuming these cells also have approximately 1 mg protein per 2.54 µL intracellular cell volume. In the same study, the glycerol content of the stratum corneum of the mouse skin (the outer most layer above the epidermis, containing flattened cells made mostly of lipids and keratin) has much higher glycerol content, approximately 5 μmol/mg protein, (ie, ∼135-fold more concentrated than the epidermis) (100). In AQP3-KO mice, a lower stratum corneum glycerol content is associated with reduced skin hydration, pointing to the importance of glycerol in the skin as a humectant (100). Unclear is whether the bulk of this skin glycerol is cytosolic, bound to membranes, extracellular, or produced by skin bacteria. The much higher level of glycerol in or around the cells of the upper skin layers likely serves as a counter-stress and moisturizing/hydrating/humectant agent to protect these cells and the body at large from extreme changes and stresses of the environmental milieu, such as air, dust, heat, cold, and osmotic pressure due to exposure to water, etc. Overall, except for the outer skin layer where the calculated cell-associated glycerol concentration appears to be in the molar range, the bulk of the examined studies reported values of intracellular glycerol of about 5 to 50 mM, and these numbers likely also include Gro3P in the extracts, as mentioned earlier.

### Crowding in Intracellular Environments and Glycerol

Considering that glycerol is transported into and out of cells via facilitative transporters, a greater than 100 magnitude of free intracellular compared to free extracellular glycerol concentration in some cell types at first seems highly unlikely, as glycerol uptake into cells could not occur. Furthermore, GK, which has a high affinity for glycerol (Km ∼5 µM) (166), would always be saturated if the intracellular free glycerol concentration were to be in the millimolar range, which is not the case. The explanation may lie in the complexity of the intracellular environment, which is crowded by macromolecules (>200 mg/mL in cytoplasm (167)), as glycerol is predicted to be in much higher concentrations within solvation layers surrounding macromolecules (bound via hydrogen bonds) than within the bulk intracellular water (as free glycerol) (168-170). The potential roles of glycerol within cells at such high concentrations (eg, protein stabilization) are discussed in the next section. Thus, clearly there are large gaps in knowledge with respect to the intracellular distribution of glycerol.

## Cellular Functions of Glycerol

**Figure 7.**
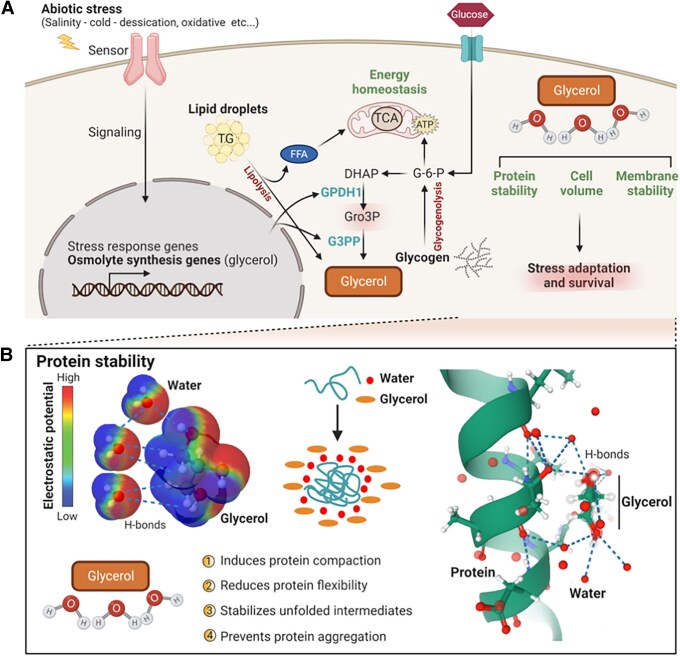
Illustration of the role of glycerol in abiotic stresses and protein stability and homeostasis. A, Stress sensors on the cell membrane or intracellularly can sense various forms of abiotic stress such as cold, heat, salinity, oxidative, and other insults to stimulate pathways and genes linked to adaptation, repair, and detoxification. Many stresses can induce enzymes involved in glycerol biosynthesis such as GPDH1 and G3PP (in *C elegans*, PGPH, homologue of G3PP). In organisms like *C elegans*, stresses like cold or excess salinity can also stimulate glycogenolysis to release glucose, which is used for glycerol production as well as energy production in mitochondria. In adipocytes cold stimulus causes lipolysis of triglycerides stored in lipid droplets resulting in the production of glycerol and FFA, which are used for energy production. Glycerol produced in the cells participates in the maintenance of protein and membrane stability and structure, and in the regulation of cell volume by acting as an osmolyte. B, Because of its ability to form hydrogen bonds and interface between macromolecules and water molecules, glycerol has the ability to maintain protein and macromolecular structure, to stabilize unfolded or misfolded proteins, and act as a chemical chaperone to prevent aggregation of proteins like mutated huntingtin. DHAP, dihydroxyacetone phosphate; FFA, free fatty acids; G3PP, glycerol-3-phosphate phosphatase; G-6-P, glucose-6-phosphate; GPDH1, glycerol-3-phosphate dehydrogenase-1; Gro3P, glycerol-3-phosphate; TCA, tricarboxylic acid cycle; TG, triglycerides. This figure was created using BioRender under license QB28NXQAZG to Elite Possik.

Because of its chemical and physical properties, glycerol plays a critical role in adaptation to diverse stresses, particularly in microorganisms and small multicellular organisms (25). In addition to its role in metabolism, glycerol can act as an osmolyte to ensure rapid adaptation to changes in osmolarity, as a chemical chaperone to assist the folding of denatured proteins during heat or chemical stresses, and as an antifreeze in cold situations (25) and in the following subsections we describe these properties of glycerol (see Figs. 1 and  7). While the importance of some of these properties of glycerol for survival may seem to be less in mammals (eg, due to the effectiveness of complex neurohormonal regulation of temperature and serum osmolality), the importance of glycerol in stress adaptation to maintain normal cellular function in larger organisms should not be overlooked.

### Organic Osmolyte

Mechanisms of adaptation to changes in osmolarity are essential for the maintenance of cellular integrity. Hyperosmotic stress causes rapid efflux of water from cells, resulting in cell shrinkage, and macromolecular damage, cell cycle arrest, and death (171, 172). Organic osmolytes, including amino acids, sugars, and polyols (sucrose, sorbitol, trehalose, mannitol, glycerol, myo-inositol), methylamines (glycine betaine, glycerophosphorylcholine, trimethylamine *N*-oxide, and others), and urea protect the cells from osmotic stress (173-177). Glycerol is a unique osmolyte that can accumulate in the cells to high concentrations to counter osmotic stress and maintain cell volume (see Figs. 1 and 7), without affecting cellular function, to ensure survival (4, 25, 177).

The mechanisms of adaptation to osmotic stress have been extensively studied in yeast and *C elegans* (78, 178, 179). In *C elegans*, following hyperosmotic stress, the animals swell, activating a signal transduction pathway that leads to acclimation and survival of the worm (179, 180). Exposure of the worms to 200 mM NaCl leads to an increase in glycerol production from glucose, from 40 to approximately 600 nmol/mg protein within the first 24 hours (179). The expression of the Gro3P dehydrogenases *Gpdh-1* and *Gpdh-2*, involved in the synthesis of glycerol from DHAP, rapidly increases in the worm during osmotic stress, whereas double mutants of *Gpdh-1 and Gpdh-2* show reduced glycerol synthesis and intolerance to osmotic stress (179, 181, 182). Moreover, worms expressing constitutively active *Gpdh-1* or with elevated glycogen stores and *Gpdh-1* andG*gpdh-2* are resistant to osmotic stress (182-184). We additionally showed in *C elegans* that the expression of the 3 G3PP isoforms (*Pgph-1*, *Pgph-2*, and *Pgph-3* genes) is increased in response to hyperosmotic stress, to enhance glycerol production and to protect against hyperosmotic stress (78). Sugars such as glycogen and trehalose are rapidly degraded under osmotic stress conditions to ensure glycerol production (183, 185, 186). Interestingly, it has been shown that exposure of mother worms to mild osmotic stress bestows osmotic protection to the offspring, likely via epigenetic alterations and expression changes in the maternal germline of genes like *Gpdh-2* that enhance the production of glycerol (186-188).

In humans, serum osmolality is tightly regulated in the range of 285 to 295 mOsmol/kg by complex thirst, hormonal (eg, antidiuretic hormone, renin-angiotensin-aldosterone system), and renal mechanisms; however, for the kidneys to concentrate urine, the renal medullary cells build up high levels of organic osmolytes that include glycerol (175, 189). Little is known about how other mammalian cells modulate intracellular osmolyte concentrations, including glycerol, to adapt to disease-related hypo-osmotic and hyperosmotic stresses.

### Protein Stability and Folding

Glycerol is predicted to be in high concentrations within solvation layers surrounding macromolecules such as proteins (bound via hydrogen bonds) (168-170), and by acting as a chemical chaperone to stabilize proteins through preventing their unfolding and aggregation during refolding (4, 25, 168, 177) (see Fig. 7). An important role of glycerol for restoring proper folding of proteins was suggested by studies showing that glycerol facilitates normal folding of cancer-related mutant TP53 proteins that are necessary for restoring sensitivity to radiation therapy (190). In another study relevant to Huntington disease, it was shown that polyol osmolytes like glycerol stabilize and promote the aggregation and compaction of mutant huntingtin (mHtt^Exon1^) and lower the cellular levels of diffuse mHtt, which otherwise binds with regulatory proteins such as cyclic adenosine monophosphate–responsive element binding protein and induces apoptosis (191). Overall, glycerol can act not only as an osmolyte but also as a protein stabilizer and a chaperone to promote normal folding of mutant proteins.

### Antifreeze and Thermal Acclimation to Low and High Temperatures

The rainbow smelt fish *Osmerus mordax*, like many other arctic fish, endures severe drops in water temperature, down to −2 °C, by avoiding freezing in part by producing antifreeze proteins and by accumulating glycerol, which lowers the freezing point to −2 °C, preventing cold denaturation of its proteins (22, 192-195) (see Fig. 7). Glycerol is constantly produced and accumulates in the plasma and tissues of the fish during cold conditions (22, 193, 194). Studies with labeled glucose revealed that most of the glycerol in these fish is produced from glycogen degradation in the muscle and liver, with some contribution from glyceroneogenesis, but not from lipolysis (192). Recently, two potential G3PP enzymes have been identified in this fish (196), and their transcripts were shown to increase after cold acclimatization, even though their role in glycerol production or enzymic activity to generate glycerol was not directly demonstrated (196, 197).

With respect to heat stress, *C elegans* exposed to 400 to 600 mM glycerol was found to be better tolerant when transferred from 20 °C to 35 °C, unlike the worms grown without glycerol (4). Furthermore, in human fibroblasts exposed to 45 °C for 30 minutes in vitro, glycerol (300 mM in cell culture) protected cells from dying and allowed their proliferation. It was proposed that this beneficial effect is due to chaperoning of proteins by glycerol itself, and by glycerol inducing proteasome activity and increasing the expression of the molecular chaperone mortalin (mtHSP70) (4). Furthermore, it has been shown that glycerol supplementation with or without creatine is an effective way for reducing thermal and cardiovascular responses by hyperhydration (198, 199).

## Clinical and Translational Implications of Glycerol in Health and Pathophysiology

**Figure 8.**
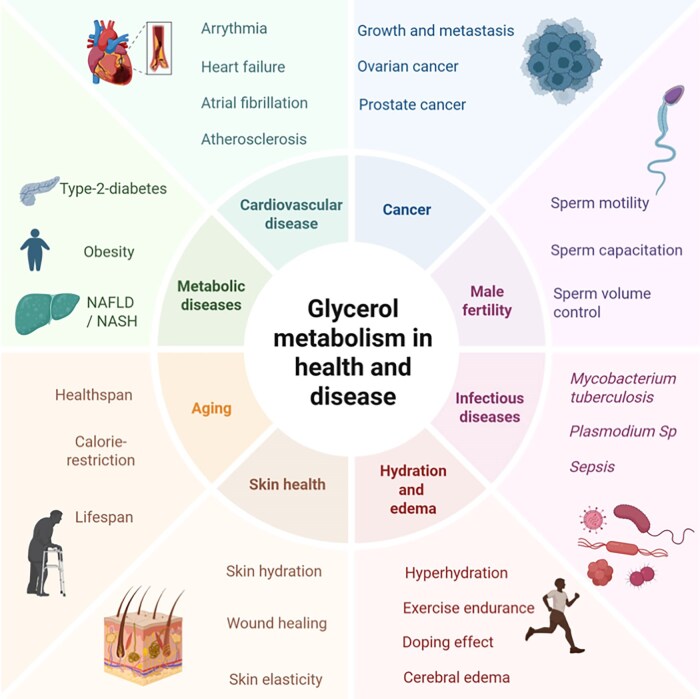
Glycerol metabolism in health and disease. Glycerol and glycerol-3-phosphate play a central role in intermediary and energy metabolism. Different pathological conditions such as cancer, infectious diseases, and cardiovascular and metabolic diseases and also cerebral edema are found to be associated with altered metabolism of glycerol and glycerol-3-phosphate. Glycerol metabolism is also implicated in spermatozoa health and male fertility and in skin hydration and wound healing. Enzymes involved in glycerol metabolism are also known to affect aging, affecting healthspan and lifespan. In addition, consumption of glycerol-containing drinks has been shown to be helpful in exercise endurance due to its hydrating effects. This figure was created using BioRender under license UR28NXP7F9 to Elite Possik.

Glycerol production and its levels in plasma, urine, and different tissues are often elevated in various pathological conditions (see Figs. 5 and  8) as discussed next.

### Obesity and Cardiometabolic Disorders

Plasma glycerol and FFA levels, likely as a consequence of increased TG lipolysis from adipose tissue, are increased in obese individuals, and women have higher glycerol levels than men (200). The causes for this sex-based difference in obesity-associated glycerol levels are not clear; however, it could relate to differences in the expression of adipose tissue AQP7 and hepatic AQP9, as shown in several mouse models of obesity (136, 201, 202). Thus, it was observed that in female, HFD-induced obese mice, adipose AQP7 expression is more elevated than in males (202), and this probably renders female mice less prone to obesity by facilitating the transport of glycerol out of WAT and other tissues to the blood. Of note, single-nucleotide variations in the *AQP7* gene were found to be associated with obesity and T2D (203).

Plasma glycerol has been associated with increased cardiometabolic disease (see Figs. 5 and 8). In a study of 3056 adults from the Framingham Heart Study cohort, the evaluation of the relation between cardiovascular health score (CVH) (composite of ideal body mass index, nonsmoking, physical activity, healthy diet, untreated and controlled total cholesterol, blood pressure, and absence of diabetes), and cardiovascular diseases, identified 3 metabolites (glycerol, cholesterol ester 16:1, and phosphatidylcholine 32:1) that mediated the association of CVH score with incident atrial fibrillation, and 7 metabolites, including glycerol, that partly mediated the association between CVH and incident heart failure (204). In another study, which included 30 000 individuals from the Copenhagen General Population Study with a follow-up for 10.7 years, the highest quartile nonfasting plasma glycerol and β-hydroxybutyrate concentrations, measured as indices of TG metabolism, were associated with increased risk of all-cause cardiovascular and cancer mortality, independent of plasma TG levels and body mass index (8). An analysis of baseline predictive value of fasting plasma samples from the CANagliflozin cardioVascular Assessment Study (CANVAS), a large cardiovascular outcomes trial of the sodium glucose cotransporter-2 inhibitor canagliflozin compared with placebo in T2D patients, showed that glycerol was a positive predictor of hospitalized heart failure (hazard ratio [HR] 2.21 [range, 1.45-3.35]), cardiovascular mortality (HR 1.81 [1.26-2.58]), and all-cause death (HR 1.64 [range, 1.22-2.18]), while an inverse relationship between plasma FFAs, possibly as a consequence of insulin treatment, and these cardiovascular outcomes was noticed (205). Nevertheless, an elevated level of circulating fatty acids, arising from either dietary intake or because of a reactive hyperadrenergic state and in conditions of obesity, is known to be associated with heart failure (206, 207).

Consistent with these findings of large human cohorts, patients admitted about 5 decades ago with myocardial infarction and complicated arrhythmias were found to have elevated plasma glycerol concentrations compared to patients with uncomplicated infarction (208). It is unknown if elevated glycerol has a pathogenic or beneficial role in the heart. Of note, the heart expresses AQP7 as well as GK, and the AQP7-KO mouse heart fails to adapt to pressure overloading (50).

Glycerol, which is required for TG synthesis, contributes to hepatic steatosis in MASLD. Hepatic AQP9 and glycerol permeability were studied in liver biopsies of obese patients undergoing bariatric surgery. Plasma glycerol levels were highest, and AQP9 expression and glycerol permeability were reduced, in obese people with T2D and greater severity of liver disease, suggesting downregulation of AQP9 is a defensive mechanism (139). Thus, it appears that prevention of an escalation in hepatic glycerol levels is likely beneficial against liver fat deposition. In line with this, we recently found that hepatic-specific deletion of G3PP in mice, in which the hepatic glycerol shunt activity is prevented, resulted in increased TG deposition and liver inflammation in chow-fed mice infused for 72 hours with glucose (84). Overall, plasma glycerol levels as a biomarker for cardiometabolic disorders merit more attention (see Fig. 8).

### Thermogenesis

While glycerol has important protective roles against temperature extremes in small organisms through its antifreeze and chemical chaperoning properties, it also has key roles in thermogenesis to maintain body temperature in response to cold in larger organisms. From studies in mice, it has been shown that cold exposure activates lipolysis, increasing the availability of FFAs for oxidation, and of glycerol together with FFAs for GL cycling both in BAT and WAT (209, 210). Mobilization of FFAs and glycerol from WAT on cold exposure has also been shown in humans (211). FFAs activate UCP1, particularly in BAT, which increases its thermogenic capacity (211, 212). Thus, UCP1 and enhanced FFA oxidation in BAT and possibly in beige adipocytes, and GL cycling, are important components of thermogenesis (23, 210).

Relevant to glycerol metabolism and the responses of BAT to cold exposure, and to some extent WAT, is the regulation of AQPs, particularly AQP7, and GK (114, 146). BAT expresses AQP7, which decreases during cold exposure and increases with diet-induced obesity (213). Considering that cold exposure enhances thermogenesis in BAT and this is accompanied by enhanced lipolysis, as well as GK expression and activity (214), a decrease in AQP7 by cold exposure probably ensures efficient recycling of glycerol to TGs in the BAT to favor energy expenditure via GL cycling. Indeed, GL cycling consumes 7 ATPs per turn and is thus thermogenic. Additionally, its lipogenesis and lipolysis segments generate metabolites that have several signaling roles (23, 55, 56). Of note, the reverse scenario of enhanced expression of AQP7 associated with diet-induced obesity in mice promotes BAT “whitening,” that is, conversion to a WAT phenotype with TG deposition, but the mechanism responsible for this process is not known (213).

### Plasma Glycerol and Infectious Diseases

Optimal nutrient requirements for microorganisms’ infectivity within the human body have been poorly studied (215). Glycerol was reported to be a preferred nutrient in broth cultures for certain infectious bacteria such as *Mycobacterium tuberculosis* (215), and indeed the glycerol-based Lowenstein-Jensen media has been used for culturing *M tuberculosis* since the 1930s (216). Recent studies have shown that elevated circulating glycerol under diabetic conditions serves as a nutrient to *M tuberculosis*, infecting the lungs and aggravating the bacterial infection and associated pathology (217). Thus, studies using streptozotocin and HFD mouse models of diabetes with 0.6-mM glycerol in plasma and infection with *M tuberculosis* showed significantly higher mortality and lung pathology compared to infected animals without diabetes. More evidence showing the importance of glycerol for *M tuberculosis* infectivity is that GK-mutant strains are less infective in T2D mice in which glycerol levels are elevated (217). However, the role of glycerol in *M tuberculosis* infection is complex, as some newer strains do not grow in glycerol-containing media (216), and emergent GK-mutant strains are resistant to antimicrobial treatment (218). There is a parallel story with the malarial parasite *Plasmodium* sp., in which diabetes also increases susceptibility to infection (219). Mutants of *Plasmodium* in GK (220) or in PGP/G3PP (221) have been found to be less potent in causing the disease than the wild-type organisms, indicating the importance of glycerol metabolism for infectivity in this protozoan. Thus, circulating glycerol levels may become important in driving certain infections, and this is likely more relevant in conditions of obesity or diabetes when plasma glycerol levels rise (see Fig. 8). Important to note is the observation that critically ill patients with sepsis often display elevated plasma glycerol, probably arising from increased adipose lipolysis (222, 223).

### Hyperhydration, Endurance Exercise, and Doping

Loss of body water during prolonged exercise can impair performance due to cardiovascular effects and increases the risk of hyperthermia (224-226). Hydration with water alone for prolonged exercise is not that helpful, as it lowers serum osmolality, which results in an increase in urine output and rapid reversal of the benefit (227). However, glycerol added to the water (up to 1.2 g/kg) increases serum osmolality, which, by acting as an osmolyte, aids the maintenance of an expanded plasma volume, and also by causing a reduction in urine output (224, 227). The latter is thought to be due to a direct effect of glycerol on the renal medullary concentrating mechanism; however, some effect of an increase in antidiuretic hormone cannot be excluded (189). Hyperhydration using oral glycerol is believed to protect blood flow in the skin for heat dissipation during exercise (228). Meta-analyses of studies show the benefit of preexercise hyperhydration for improvement in cardiovascular function, temperature control, and some parameters of exercise performance (224-226) (see Fig. 8). A recent study did not find an improvement in a 5-km running time trial in heat by preexercise hyperhydration using glycerol, possibly due to the countereffects of greater body mass from water retention, indicating that the effects may vary according to the type of exercise (229). Due to its hemodilution effect, glycerol hyperhydration was used by elite athletes to mask doping, which resulted in a World Anti-Doping Authority ban on this practice in 2010 (158). However, with a better understanding of glycerol's effects (153) and the recognition that it may have only minor effects on blood volume and other parameters of the Athlete Biological Passport, the ban was lifted in 2018 following further investigation (223). Ingesting sodium salt with water is another method of hyperhydration; however, using a combination of sodium and glycerol may be even more effective (230).

### Brain Edema

Brain edema is common in neurological conditions such as TBI, hemorrhagic and ischemic stroke, brain tumors, viral encephalitis, Reye syndrome, and during the treatment of DKA (231, 232). It is an abnormal accumulation of intracellular and interstitial water in the tissues of the brain. As it results in brain swelling within the fixed confines of the cranium, it increases intracranial pressure (ICP), impairs cerebral blood flow and oxygenation, and can cause brain stem herniation and death (231, 232). As an osmolyte, glycerol has been implicated in the pathophysiology of cerebral edema, but also in its treatment (3, 141, 233).

Studies on preclinical models and in patients with accidental injuries have shown that cerebral extracellular and intracellular glycerol concentrations markedly increase after TBI and stroke, in association with increased expression of AQP9 (see Figs. 5 and 8) under the transcriptional control of hypoxia-induced factor-1α (HIF-1α) (141, 161). The expression of AQP4, which transports water but not glycerol, is also increased under the regulation of HIF-1α, and is considered a pharmacological target to prevent edema in TBI (141, 234). Lipolysis of brain phospholipids may also increase glycerol in response to brain injury (163). As an osmolyte, elevated glycerol in the intracellular and interstitial fluid compartments of the brain attracts water, contributing to edema.

Intravenous infusion of hypertonic fluids containing saline, mannitol, or glycerol, sometimes in combination, are also used therapeutically to reduce cerebral edema to lower ICP by increasing the intravascular to interstitial fluid osmotic gradient (232, 233, 235). All are effective in reducing ICP in the short term, but there is debate about which should be used and in which patients (17). Of note, a recent meta-analysis suggested that compared to mannitol, glycerol has similar effectiveness in the management of cerebral edema, but a better safety profile (233). The level of evidence for long-term neurological outcomes of the use of hypertonic fluids, including glycerol and/or mannitol, is low (17, 233). A larger trial of continuous infusion of hypertonic saline in moderate to severe TBI patients showed no improvement in neurological outcomes after 6 months (235). A concern with glycerol administration is rebound brain edema, particularly if the blood-brain barrier is disturbed. In a case report of a 25-year-old man with a severe TBI, microdialysis monitoring in the frontal lobe showed a marked increase in interstitial fluid glycerol following treatment with an 85% glycerin suppository, increasing the risk of aggravating brain edema (236).

An improved understanding of the pathophysiology of cerebral edema is needed if targeted therapies are to be developed, not only in conditions such as TBI and stroke, but also in metabolic conditions such as DKA and Reye syndrome. The potential role of intracellular glycerol regulation in the brain, as an osmolyte and chaperone, in response to hypo-osmolar and hyperosmolar stresses also needs investigation. In addition, it remains uncertain if glycerol administration in the management of cerebral edema is beneficial or harmful.

### Cancer

For cancers to grow and metastasize, their cells need a constant source of nutrients to support biosynthesis of all cellular components, including nucleic acids, proteins, and complex lipids, often within nutrient-poor and hypoxic environments (237, 238). To achieve this, cancer cells adapt their metabolic pathways according to the cancer cell's origin, the stage of oncogenesis, the tumor microenvironment, as well as changes in whole-body metabolism. Cancers also require adaptations in bioenergetic mechanisms to sustain increased cell energy requirements without succumbing to oxidative stress (238). Considering the central role of glycerol in metabolism, it should be no surprise that evidence of modifications in all pathways of glycerol metabolism, as well as AQP expression (239), has been documented in various cancers (see Figs. 5 and 8).

One hundred years ago, Otto Warburg reported that cancer cells show markedly increased glucose uptake and lactate production, even in the presence of oxygen, called “the Warburg effect,” which is active in the majority of cancers (240, 241). Markedly accelerated glycolysis with lactate production in cancer cells can be a source of ATP with nicotinamide adenine dinucleotide (NAD^+^) replenishment even in hypoxic environments and also provides intermediates for nucleic acid, nonessential amino acid, and hexosamine synthesis, as well as creating an acidic tumor microenvironment favoring cancer growth and metastasis (241-243). The lactate produced by the tumor provides a nutrient to normoxic cancer cells and noncancerous cells within the tumor microenvironment, and reduces immune attack on cancer cells (241-243). The increased flux through glycolysis provides a source of Gro3P necessary for GL synthesis, particularly membrane phospholipids, essential for cancer cell growth and survival (237, 244-246).

In addition to lactate being an exit point of carbons from glycolysis with recovery of NAD^+^, so is the “glycerol shunt” via GPDH1-mediated reduction of DHAP to Gro3P, and then dephosphorylation of Gro3P by G3PP. We recently showed that G3PP expression is elevated in aggressive prostate tumors, being strongly associated with recurrence, progression, bone metastasis, and increased cancer-specific mortality (see Fig. 5) (12). Accordingly, we proposed that elevated G3PP is an independent biomarker for the prediction of prostate cancer bone metastasis (12). Like lactate production, the possibility exists that glycerol production via G3PP would lessen mitochondrial oxidative burden, alter the tumor microenvironment, and provide an alternate source of nutrients to other surrounding cancer and noncancerous stromal cells. Here, we propose to name this glucose-derived glycerol plus lactate production by cancer cells the “neo-Warburg effect.” Indeed, glycerol has recently been proposed as a nutrient for castration-resistant human prostate cancer PC3 cells with a capacity to promote xenograft tumor growth in mice (247). Noteworthy is a recent report showing reduced xenograft tumor growth in nude mice with G3PP-deleted HeLa cells; surprisingly, this study also noticed a reduction in tumor growth with G3PP overexpressing HeLa, A549 human lung carcinoma, and Sk-Hep1 human hepatic adenocarcinoma cells (86). These authors also showed that under hypoxia (0.5% O_2_), several types of cancer cells alter their metabolism and increase glycerol synthesis and release, a process that consumes NADH, and involves the action of aldolase B in conjunction with GPD1, GPD1L, and G3PP (86). Manipulation of glycerol synthesis and release by either increasing or decreasing the expression of any of these enzymes reduced the viability of HeLa and A549 cells under hypoxic or low nutrient conditions and the growth of corresponding xenograft tumors in mice (86). Thus, it appears that a fine balance of the glycerol shunt operation and control of cellular reductive stress by G3PP is necessary for the proliferation of tumor cells. Furthermore, it was reported using The Cancer Genome Atlas database information that among several different cancers, the survival of prostate cancer patients is adversely correlated to the expression level of G3PP and the other enzymes of the glycerol shunt (86).

Evidence also points to modulations of the Gro3P shuttle in cancer cell survival, with upregulation of mitochondrial GPDH2 believed to be involved in cancer progression (see Fig. 5) (65, 248-251). Linking the importance of Gro3P shuttle activity and the glycerol shunt in cancer metabolism is the observation that the suppression of PGP/G3PP expression in HAP1 human myeloid leukemia cells, HCT116 human colorectal cancer cells, and A375 human melanoma cells, increasing the availability of Gro3P for the Gro3P shuttle, highly sensitizes these cells to mitochondrial electron transport blockers such as pyrvinium (252). Significance of the Gro3P shuttle has also been investigated in hepatocellular carcinoma (HCC) in the context of its resistance to sorafenib (253). This study on the molecular basis of resistance to sorafenib, a multikinase inhibitor, suggested that sorafenib promotes an increased diversion of glucose carbons toward DHAP and D-lactate via methylglyoxal (MGO) production in the sensitive cells (253). However, sorafenib-resistant cells increase the production of Gro3P from DHAP and also via glyceroneogenesis and the formation of glycerol. Indeed, it was noticed that plasma levels of D-lactate are elevated in sorafenib-responder HCC patients whereas plasma glycerol levels are higher in nonresponders (253). The authors indicated that the elevated glycerol arises from the lipolytic pathways (253), but the possibility of an elevated glycerol shunt was not examined, and this possibility should be considered as information from The Cancer Genome Atlas database indicates elevated expression of G3PP in HCC. Thus, elevated plasma glycerol levels may be a biomarker for sorafenib resistance in HCC patients.

Changes in glyceroneogenesis and GK expression, both alternate sources of Gro3P to glycolysis, have been implicated in cancer. Many cancer cells express mitochondrial PEPCK at high levels and conduct glyceroneogenesis, particularly under conditions of low nutrient availability, to also produce Gro3P for the synthesis of glycerophospholipids required for membranogenesis and proliferation (254, 255). With respect to GK, cancer tissue expression of GK-5 (both mRNA and protein), as well as elevated exosomal GK-5 mRNA levels in plasma, were reported in patients with gefitinib-resistant compared to gefitinib-sensitive lung adenocarcinoma (256). Elevated GK expression has also been linked to poor prognosis, and is a proposed prognostic biomarker and therapeutic target, in patients with esophageal carcinoma (257).

The fatty acid supply to cancer cells for the synthesis of complex lipids, but also the generation of signaling molecules by GL cycling, has been shown to be important for cancer cell survival (244, 258, 259). We have shown that serum-free survival is increased in breast cancer cell lines by the provision of oleate, most evident in cell lines that are more adept at accumulating lipid droplets and have active GL cycling (258). Accumulation of lipid droplets is associated with chemoresistance in some cancers, including colorectal cancer (260).

Glycerol-transporting AQPs are elevated in different types of cancers (239, 261, 262) (see Fig. 5) and appear to facilitate metastasis (263). Thus, elevated expression of AQP3 has been implicated in the progression of tumorigenesis in skin, liver, prostate, colon, lung, and pancreatic cancers (262), and it was suggested that glycerol imported via AQP3 in the tumor cells is effectively used as a nutrient to generate ATP required for cell proliferation (264). Similarly, AQP9 has been implicated in the progression of renal cell carcinoma and glioblastoma, and both AQP7 and AQP9 are highly expressed in ovarian cancer cells (262) (see Fig. 5).

Overall, the evidence indicates that glycerol, Gro3P, and their metabolism have predominantly positive effects on cancer cell proliferation and tumor progression, depending on the type of cancer and the expression level of the different glycerol and Gro3P metabolism enzymes. These pathways are increasingly being considered in the development of cancer therapies.

### Skin Health

The skin forms a water-impermeable barrier that protects the body from the outside environment, including chemicals, microorganisms, and UV light. Increasing glycerol content of the stratum corneum toward the skin surface is critical to the maintenance of its hydration, barrier function, and healing (9, 98, 100). Accordingly, glycerol has long been used in cosmetic skin applications as a moisturizer, for example, creams to treat dry skin conditions such as psoriasis and eczema (9). Lipolysis-generated glycerol and its transport by AQP3 in the epidermal basal and spinosum layers are required to build the very high glycerol content of the stratum corneum (98, 265, 266). Another source of glycerol for skin is the lipolysis of TGs in sebaceous glands (267). Deletion of AQP3 in mice lowers glycerol content, decreases stratum corneum hydration, slows wound healing, and reduces skin elasticity, defects that can be lessened by oral glycerol supplementation, consistent with the importance of AQP3 and glycerol in the maintenance of skin health (96, 262, 265). Altered AQP3 expression has been linked to skin disorders, including psoriasis, nonmelanoma skin cancer, and atopic eczema (98) (see Figs. 5 and 8). Increasing the expression level of AQP3 by pharmacological agents such as 18β-glycyrrhetinic acid was found to improve impaired wound healing (268), and some agents like glycolic acid and all transretinoic acid were shown to protect against UV-radiation induced photoaging of the skin, possibly by upregulating AQP3 in keratinocytes (262). However, elevated AQP3-associated accelerated epidermal cell proliferation may lead to diseases such as psoriasis and possibly skin tumor growth (266). However, whether the glycerol transport function per se by either AQP7 or AQP9 is relevant for their role in skin is not established. Thus, more work is needed to clearly establish the clinical importance of AQPs, in particular AQP3, as targets for skin hydration–related diseases and to dissect the roles of other AQPs in the skin (see Figs. 5 and 8).

### Sperm Health and Male Infertility

For spermatozoa to reach their destination for fertilization, they need to sustain high rates of ATP production for motility, while coping with changes in extracellular osmolality and nutrient availability (10, 269, 270). In the upper female reproductive tract, they undergo a process of capacitation to make them competent to fertilize the oocyte (269). Glycerol may have important roles in sperm as an osmolyte and within metabolic pathways of energy metabolism critical for successful fertilization (see Figs. 5 and 8) (10). Male infertility is increased in association with obesity and T2D, so improved understanding of metabolic influences on spermatogenesis and sperm health is required (271).

Extracellular osmolality drops from 480 mmol/kg for posttesticular spermatozoa in the corpus epididymis to 294 mmol/kg in seminal fluid following ejaculation, to 268 to 284 mmol/kg in the cervical mucus, which has implications for sperm cell volume regulation (129, 272). Blockade of volume regulation in ejaculated human spermatozoa reduces swimming velocity and inhibits the penetration and migration through surrogate cervical mucus, and defective sperm volume regulation was reported to be related to infertility in men (272). AQP3, -7, -8, and -11 are all present in sperm, with the expression levels/activity of AQP3 and AQP7 being shown to be reduced in asthenozoospermic individuals as a possible cause of infertility (129, 273-276). Thus, the regulation of spermatozoa glycerol content for sperm volume control may be of importance, warranting further investigation (see Figs. 5 and 8).

With respect to metabolism in spermatozoa, glycolysis and mitochondrial oxidative phosphorylation are both considered important, including during capacitation when energy requirements increase (269, 270). The roles of glycerol metabolism are less clear, but glycerol uptake, the Gro3P shuttle, and the glycerol shunt, as discussed next, are all likely to be important. Supporting a role for glycerol uptake, bull spermatozoa were found to metabolize ^14^C-glycerol in vitro (3). Among the GK family, glycerol kinase-like protein GK2 and glycerol kinase-like-1 (GKl1) are expressed in the murine testis, and GK2 in human testis (277, 278). GK2 and GKl1 genes have both arisen by autosomal transposition of the *GK* gene present on the X chromosome (279). Neither GK2 nor GKl1 has glycerol kinase activity in vitro (28); however, their deficiency in mice causes disordered mitochondrial sheath formation in sperm and male infertility (277, 278).

Spermatozoa can produce Gro3P from glucose or fructose, phospholipid degradation, and breakdown of glycerophosphocholine (GPC) (280, 281). GPC has also been shown to be important for energy metabolism and capacitation in rat and human sperm (282, 283). Spermatozoa express a testis-specific form of GPDH2, with a restricted expression in postmeiotic germ cells (284), and to detect Gro3P originating from GPC, it is necessary to inhibit GPDH2, suggesting that Gro3P is rapidly used by GPDH2 in the Gro3P shuttle (280, 285). GPDH2-KO mice have impaired sperm ROS production associated with impaired capacitation, indicative of the importance of the Gro3P shuttle for normal spermatozoa function (286).

G3PP is expressed at very high levels in testis and spermatozoa (6), and is used to generate glycerol (287). A quantitative proteomics study of sperm from normal men and testicular seminoma patients with reduced fertility identified 393 differentially expressed proteins, among which G3PP expression was found to be reduced by approximately 50-fold in the seminoma sperm (288). We recently showed that G3PP expression markedly increases during mouse spermatogenesis (287). Male mice with germ cell G3PP deletion from the embryonic stage are infertile due to dysfunctional sperm with reduced motility and capacitation, elevated spontaneous acrosomal reaction, and oxidative stress. As the sperm of conditional G3PP-KO mice release less glycerol and show elevated Gro3P and ROS levels and increased mitochondrial membrane potential, the mechanism of sperm dysfunction is likely related to a rise in Gro3P that drives more electrons to the electron transport chain via GPDH2 and the Gro3P shuttle, with the resultant hyperpolarization of the mitochondria and excessive ROS production (287). In agreement with this possibility, while optimal ROS generation is necessary for sperm capacitation, excess ROS production leads to male infertility (289). Other potential roles of G3PP and the glycerol shunt in sperm are the replenishment of NAD^+^ to support glycolysis and to provide glycerol as an osmolyte. Thus, G3PP and the glycerol shunt are essential for male fertility (287). Another source of glycerol is through the lipolysis of GLs. Of note, deficiency of hormone-sensitive lipase is associated with male sterility (290). Overall, the available information indicates that spermatozoa can produce glycerol and also use glycerol by employing distinct pathways and enzymes. Glycerol itself and/or via its metabolism by GK or the glycerol shunt, and Gro3P as well, play a key role in sperm health and male fertility, as any dysregulation of glycerol and Gro3P metabolism causes disturbed sperm function and male infertility via mechanisms that need to be understood.

### Aging and Glycerol Shunt

Evidence linking excess nutrient supply, particularly of simple sugars, to the process of aging is strong (95, 291), while the evidence points to low glycemic index diets, calorie restriction, and intermittent fasting being protective (291). A reduced NAD^+^/NADH ratio, oxidative stress with ROS production, buildup of advanced glycation end products (AGEs), and altered autophagy, have all been implicated in the aging process (94, 95, 292, 293). The glycerol shunt, which diverts glycolytic intermediates such as DHAP and GA3P away from MGO production, and from mitochondrial metabolism, while increasing cellular NAD^+^, could therefore be protective of aging (94, 294-296), with evidence for this being the case in *C elegans* (297). MGO is a highly reactive dicarbonyl compound that drives glycation of macromolecules and AGE production (94, 95, 292, 297). A higher NAD^+^/NADH ratio in cells reduces oxidative stress and increases the activity of sirtuins, which are antiaging (95, 298, 299). By reducing the availability of Gro3P for the mitochondrial Gro3P electron shuttle, the glycerol shunt should reduce the production of ROS and AGEs, and maintain a high NAD^+^/NADH ratio, thereby protecting against telomere damage and senescence (300).

Cellular senescence plays a dual role during aging. It protects against cancer but, with age, senescent cells accumulate and release inflammatory factors. This disrupts tissue function, impairs regeneration, and drives organismal aging and age-related diseases. A recent study provided strong evidence for a role of Gro3P, GK, and G3PP in the regulation of cellular senescence. Thus, it was noticed that preventing the accumulation of Gro3P either by overexpressing G3PP activity or by inhibiting GK has senomorphic effects in human fibroblasts and myoblasts exposed to different senescence-inducing agents (301). Though the exact mechanism is not clear, it was suggested that controlling lipid droplet biogenesis by lowering TG synthesis likely interferes with cellular senescence (301).

Our recent work on *C elegans* strongly supports the view that G3PP activity and the glycerol shunt play an important role in the healthspan and lifespan of this organism, particularly under metabolic stress/glucotoxicity (see Fig. 8). Thus, we noticed that downregulation of G3PP/PGP homologues in the worm led to reduced glycerol production, increased susceptibility to osmotic stress and glucotoxicity, and decreased healthspan (measured as movement of the worm) and lifespan, whereas elevated expression of the G3PP/PGP homologues had the opposite effects improving the worm's healthspan and lifespan (11, 78). These effects of increasing the glycerol shunt in the worm were found to be related to enhanced autophagy flux (11). As far as the mechanism is concerned, G3PP/PGP homologue overexpression and increased glycerol shunt activity counter metabolic stress and promote healthy aging in *C elegans* via a glycogen depletion sensing-AMP-kinase-HLH-30/TFEB-autophagy axis (11). Interestingly, overexpression of the PGPH homologue in *C elegans* under glucotoxic conditions extends healthspan and partially mimics the beneficial effects of calorie restriction without altering food intake (pharyngeal pumping) or fertility, through a novel, noncanonical dietary restriction and non–insulin-like mechanism (11, 78). This G3PP-mediated glycerol shunt may function as a calorie restriction mimetic pathway independent of food intake. Further research in higher organisms is needed to assess the role of G3PP in aging.

These findings of altering G3PP/PGP homologue expression in *C elegans* are consistent with our findings of mammalian cells in vitro and in mice. We have shown that G3PP and the glycerol shunt play an important role in the protection from glucotoxicity in rat INS-1(832/13) β cells (6), mouse islet β cells (85), and hepatocytes (84). Thus, it is plausible that enhancing the glycerol shunt/G3PP activity can be harnessed to tackle an important root cause (nutrient excess) and the core signaling pathways involved in multiple aging-associated diseases (see Fig. 8).

### Acute Kidney Injury, Bone Metabolism, and Fibroblast Growth Factor-23

Lipid metabolism plays an important role in bone development. Defects in LPA metabolism lead to sternal and costal abnormalities as well as a decrease in bone mass (302, 303), which highlights a role of LPA in bone remodeling. Recent findings suggest that Gro3P in circulation contributes to elevated GPAT2-mediated LPA synthesis in osteocytes in bone marrow, leading to enhanced fibroblast growth factor-23 (FGF23) production (304, 305). Importantly, kidney production of Gro3P and its release into the renal vein increases in acute kidney injury cases. Increased levels of FGF23 lower kidney proximal tubular reabsorption of phosphate and also reduce the synthesis of kidney 1,25(OH)_2_D_3_ and intestinal phosphate uptake (306). However, the precise mechanism of Gro3P-mediated effects on bone development and bone remodeling remains largely unknown (307). In addition, how circulating Gro3P may enter cells and is used by the GPAT2 of bone marrow mesenchymal cells to generate LPA is not clear. Considering that G3PP regulates lipid metabolism (7, 84), and bone marrow adipocyte lipid metabolism plays a role in bone homeostasis, it is interesting to investigate the role of G3PP in the regulation of bone marrow homeostasis and metabolism.

### Glycerol-3-Phosphate Activation of Carbohydrate Response Element Binding Protein: Role in Citrin Deficiency and in Ethanol Metabolism

Genetic defects of citrin (SLC25A13), a mitochondrial aspartate/glutamate transporter-2 that is associated with an aversion to carbohydrate-rich foods and fatty liver, causes elevated urinary excretion of glycerol and Gro3P in affected patients and in a mouse model of citrin deficiency (160, 308). Hepatic Gro3P levels were also found to be elevated in the mouse model (308). Two independent recent studies showed that Gro3P binds and activates a central transcription factor of metabolism, ChREBP, which promotes the transcription and synthesis of FGF21 (34). This is particularly interesting as FGF21 is a novel target for cardiometabolic disorders (309). It was shown that enhanced activation of ChREBP and elevated synthesis of FGF21 in citrin-deficient patients is responsible for the aversion to sugar-rich foods and perhaps fatty liver (34). Furthermore, ethanol, by increasing NADH in the liver, increases Gro3P levels, which in turn lead to elevated synthesis of FGF21, which acts centrally to elicit a protective response against ethanol toxicity (34). Considering that ChREBP is also involved in the proliferation of some cancer cells, it is of interest to examine if Gro3P plays a role in the regulation of this transcription factor to alter cancer growth.

## Conclusion and Perspectives

**Figure 9.**
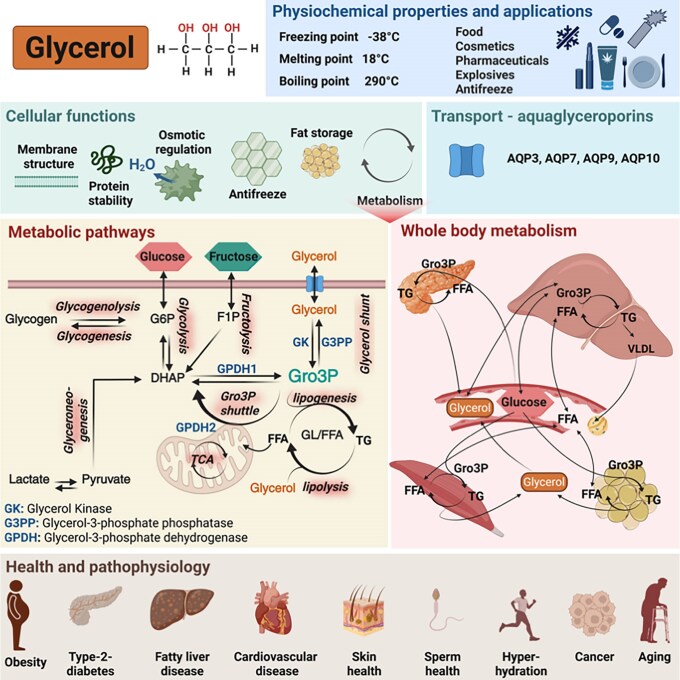
Overview of glycerol and its metabolism in health and diseases. The figure summarizes key aspects of glycerol metabolism. AQP, aquaporin; DHAP, dihydroxyacetone phosphate; F1P, fructose-1-phosphate; FFA, free fatty acids; G3PP, glycerol-3-phosphate phosphatase; G6P, glucose-6-phosphate; GK, glycerokinase; GL, glycerolipid; GPDH1, glycerol-3-phosphate dehydrogenase-1; GPDH2, glycerol-3-phosphate dehydrogenase-2; Gro3P, glycerol-3-phosphate; TCA, tricarboxylic acid cycle; TG, triglyceride; VLDL, very low-density lipoprotein. This figure was created using BioRender under license BC28NXQGMI to Elite Possik.

In this review on glycerol in metabolism, health, stress, and disease, we have covered its unique physicochemical properties as an osmolyte and chaperone of macromolecules and its central role in whole-body metabolism, including the enzymes and AQPs involved in its metabolism and flux in and out of cells. Its importance to the maintenance of health of various organs and its involvement in a wide range of pathologies, including cardiometabolic disease, skin disorders, tissue hydration, cancer, infertility, and aging, is discussed (Fig. 9). Our recent discovery of the G3PP enzyme that releases free glycerol from Gro3P, enabling nutrient detoxification via a pathway we have called “the glycerol shunt,” has been presented in more detail.

While the importance of glycerol's physicochemical properties to the survival of microorganisms and small multicellular organisms to osmotic and temperature stress (eg, in *C elegans,* yeast, and rainbow smelt) is well established, less is known about how these properties affect cellular health in larger organisms. The seemingly very high concentrations of intracellular glycerol in mammalian cells need to be verified by alternate reliable methods, with likely roles being stabilization and protection of macromolecules. The physicochemical properties for skin health are clearly important, which underpins its extensive use both in cosmetic and therapeutic skin products. Its importance as an osmolyte for sperm health warrants further investigation.

Tissue-specific glycerol metabolism and glycerol flux between tissues is highly regulated by AQPs and enzymes. A greater understanding of how glycerol metabolism contributes to the overall body energy metabolism and thermogenesis is needed to better appreciate its role in the maintenance of cardiometabolic health. It is important to note that cellular glycerol likely regulates the expression of certain genes of its own metabolism, but whether the effect is direct or via a derived metabolite is unknown. Glycerol regulation of gene expression is thus an area that is worth exploring, considering the recent studies showing the activation of ChREBP transcription factor by Gro3P. G3PP activity and the glycerol shunt, by enabling cellular exit of excess glucose carbons in the form of glycerol, are pathways by which the production of AGEs and MGO, and the oxidative burden on cells, can be reduced. Hence, G3PP is a promising therapeutic target to prevent cardiometabolic disease and to promote healthy aging. In some cancers, the expression of G3PP is increased, and we have proposed “the neo Warburg effect” in which, in addition to glycolysis being upregulated, activity of the glycerol shunt is also increased. Thus, G3PP and glycerol metabolism could be targeted in cancer therapeutics. G3PP is highly expressed in the testis, and further exploration of its role, and of glycerol metabolism more broadly, in sperm health and fertility is warranted.

In conclusion, although glycerol is a relatively simple molecule, its importance in health and disease is underrealized. There is much more to be learned. For these reasons, we hope this review stimulates much interest and ongoing investigation into glycerol, its metabolism, and its roles in health and disease, and into glycerol-related metabolic enzymes and pathways as targets for therapy.

## Abbreviations

AGE: advanced glycation end product
AQP: aquaporin
ATP: adenosine triphosphate
BAT: brown adipose tissue
ChREBP: carbohydrate response element binding protein
CVH: cardiovascular health score
DHAP: dihydroxyacetone phosphate
DKA: diabetic ketoacidosis
F1P: fructose-1-phosphate
FFAs: free fatty acids
FGF21: fibroblast growth factor-21
FGF23: fibroblast growth factor-23
GA3P: glyceraldehyde-3-phosphate
G3PP: glycerol-3-phosphate phosphatase
G6P: glucose-6-phosphate
GK: glycerokinase
GL: glycerolipid
GPC: glycerophosphocholine
GPD1L: glycerol-3-phosphate dehydrogenase-1L
GPDH1: glycerol-3-phosphate dehydrogenase-1
GPDH2: glycerol-3-phosphate dehydrogenase-2
Gro3P: glycerol-3-phosphate
HCC: hepatocellular carcinoma
HFD: high-fat diet
HIF-1α: hypoxia-induced factor-1α
ICP: intracranial pressure
KO: knockout
LPA: lysophosphatidic acid
MAGs: monoglycerides
MASLD: metabolic dysfunction–associated steatotic liver disease
MGO: methylglyoxal
mRNA: messenger RNA
NAD^+^: nicotinamide adenine dinucleotide
PEPCK: phosphoenolpyruvate carboxykinase
PPARγ: peroxisome proliferator-activated receptor γ
ROS: reactive oxygen species
T2D: type 2 diabetes
TBI: traumatic brain injury
TCA: tricarboxylic acid cycle
TGs: triglyceride
VLDL: very low-density lipoprotein
WAT: white adipose tissue
